# Tailoring Syringic Acid–Trimesic Acid Mixed-Linker MIL-100(Fe): Evaluation of Drug-Loading Capacity, Bioavailability, and Toxicity

**DOI:** 10.3390/pharmaceutics18030309

**Published:** 2026-02-28

**Authors:** Joshua H. Santos, Hannah Jean Victoriano, Mary Sepulveda, Hung-En Liu, Shierrie Mae N. Valencia, Rikkamae Zinca Marie L. Walde, Emelda A. Ongo, Chia-Her Lin

**Affiliations:** 1 Department of Science and Technology—Central Office, General Santos Avenue, Upper Bicutan, Taguig City 1631, Philippines; 2 Department of Science and Technology, Industrial Technology Development Institute, General Santos Avenue, Upper Bicutan, Taguig City 1631, Philippines; 3 Department of Chemistry, National Taiwan Normal University, No. 162, Section 1, Heping E Rd, Da’an District, Taipei City 10610, Taiwan; 4 Department of Chemistry, National Tsing Hua University, 101, Section 2, Kuang-Fu Road, East District, Hsinchu 300044, Taiwan

**Keywords:** syringic acid, bioavailability, MIL-100(Fe), mixed-linker

## Abstract

**Background/Objectives:** The use of the drug delivery system is notable for the systemic improvement of low orally bioavailable compounds, such as the bioactive phenolic acid, syringic acid. Innovative techniques are employed to enhance the performance of certain drug delivery systems. In connection with our previously reported journal with the use of MIL-100(Fe) as a drug carrier for syringic acid, this study utilized a mixed-linker synthesis of syringic acid and trimesic acid and characterized the properties in comparison with the unmodified MIL-100(Fe) through a solid solution approach. **Methods:** Modified MIL-100(Fe) was synthesized by substituting different molar concentrations of syringic acid for trimesic acid through de novo synthesis. Simple impregnation of syringic acid was carried out at 12, 24, 36, and 48 h and at 1:1 and 1:2 molar ratios of MIL-100(Fe) to syringic acid. Characterization was performed via PXRD, FTIR, BET, SEM, and DLS. In vivo studies included acute oral toxicity testing (OECD 425) and bioavailability assessment in Sprague Dawley rats. **Results:** The optimized amount of syringic acid to be substituted for trimesic acid is 0.10 mmol, as confirmed by the value of the PXRD. Optimized drug loading of 66.85 ± 0.004% was achieved using a 1:2 ratio of syringic acid to MIL-100(Fe)-10% over 36 h. Structural modifications were confirmed via FTIR, specifically through shifts at 1239.2 cm^−1^, while TGA demonstrated thermal stability up to approximately 350 °C. Morphological analysis by SEM showed octahedral particles (210.70 ± 1.23 nm), and a decrease in BET surface area post-loading verified successful encapsulation. While in vitro release was media-dependent, toxicity studies at 2000 mg/kg showed no adverse effects; notably, SGOT and SGPT levels decreased, though BUN and creatinine levels rose. Compared to pure oral syringic acid, the SYA@MIL-100(Fe)-10% formulation demonstrated a 5.09-fold increase in relative bioavailability. Furthermore, it outperformed intraperitoneal administration of the drug by 1.65-fold. **Conclusions:** Modification of MIL-100(Fe) by incorporating syringic acid into the framework as a substituted organic linker indicates that SYA@MIL-100(Fe)-10% is a safe and effective delivery system for syringic acid, enhancing oral bioavailability. To the best of our knowledge, this is the first study to investigate the mixed-linker synthesis of MIL-100(Fe) by utilizing syringic acid as a structural co-ligand, rather than solely as an encapsulated guest. While MIL-100(Fe) has been extensively employed as a carrier for various therapeutics, this research uniquely integrates the active agent into the framework lattice itself to modulate porosity and loading capacity, subsequently evaluating its systemic performance in an in vivo model.

## 1. Introduction

Drug delivery systems are critical for enhancing the therapeutic efficacy of bioactive compounds by modulating their pharmacokinetics, improving solubility, and overcoming physiological barriers [1]. Among the various platforms developed, metal–organic frameworks (MOFs) have emerged as a superior class of hybrid porous materials. Composed of metal nodes connected by organic linkers, MOFs offer exceptionally high surface areas, tunable pore sizes, and structural biodegradability, making them ideal candidates for encapsulating challenging drug molecules [2].

**Figure 1:**
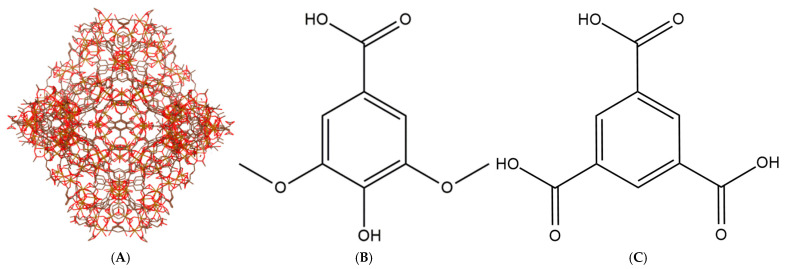
Chemical structures of (**A**) MIL-100(Fe), (**B**) syringic acid (3,5-dimethoxy-4-hydroxybenzoic acid), and (**C**) trimesic acid (benzene-1,3,5-tricarboxylic acid) (as visualized using VESTA, version 2025, Japan).

**Figure 2:**
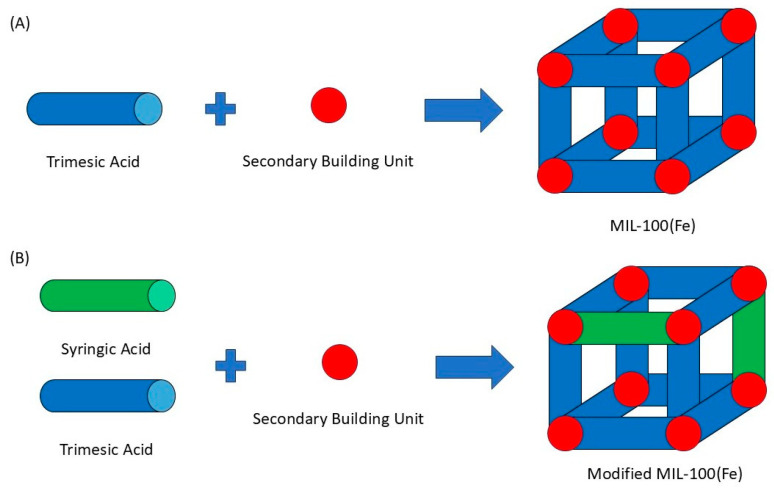
Graphical illustration of the formation of (**A**) MIL-100(Fe) from the reaction of trimesic acid and a secondary building unit (Iron [III]), and (**B**) modified MIL-100(Fe) from the reaction of syringic acid, trimesic acid, and a secondary building unit (Iron [III]) (as visualized in Microsoft PowerPoint 2021).

In particular, the iron (III) carboxylate framework, MIL-100(Fe) (Materials of Institute Lavoisier) (Figure 1A), has garnered significant attention due to its biocompatibility, large pore volume, and stability in aqueous media. The synthesis of MIL-100(Fe) is driven by a Lewis acid–base reaction, wherein trimesic acid (Figure 1C) serves as the nucleophilic organic linker coordinating to the metal centers (as depicted in Figure 2A). Structurally, the resulting framework contains two distinct mesoporous cages accessible through microporous windows. However, while the pristine framework is effective, its native pore environment may not always offer the optimal interaction for specific guest molecules [3,4,5].

To address this, mixed-linker strategies have been developed to tailor framework properties. By incorporating a secondary organic linker during synthesis, researchers can introduce structural defects, modulate pore aperture size, and alter the chemical environment of the pores without destroying the overall topology [6,7].

Building upon our previous work [8], which established MIL-100(Fe) as a viable carrier for syringic acid (SA), this study explores a de novo mixed-linker approach. Unlike trimesic acid (Figure 1C), which is tritopic (possessing three carboxylic acid groups), syringic acid (Figure 1B) is monotopic and possesses unique nucleophilic moieties (methoxy and hydroxyl groups) [9]. We hypothesized that substituting a portion of the trimesic acid with syringic acid would not only functionalize the framework but also engineer structural defects and facilitate specific iron–phenolate coordination to enhance drug-loading capacity (Figure 2B). Syringic acid itself is a potent phenolic compound with antioxidative, anti-inflammatory, and anti-diabetic properties [10], but its clinical utility is severely hampered by low oral bioavailability (approx. 32.48% relative to the intraperitoneal route).

Consequently, this study aims to develop a modified MIL-100(Fe) carrier employing a dual-incorporation strategy. Mixed-linker MIL-100(Fe) frameworks were synthesized by varying the molar ratio of syringic acid to the standard trimesic acid linker, followed by physical loading of additional syringic acid into the pores via simple impregnation. The physicochemical properties of the resulting carriers were characterized using powder X-ray diffraction (PXRD), particle size analysis, surface morphology (SEM), Brunauer–Emmett–Teller (BET) surface area analysis, and FT-IR. In vitro release profiles were evaluated in simulated gastric (pH 1.2), intestinal (pH 6.8), and physiological (pH 7.4) fluids, with release kinetics analyzed using five mathematical models. Safety was assessed according to OECD Guideline 423, and systemic performance was evaluated in a Sprague Dawley rat model by comparing the area under the curve (AUC) of the modified carrier against free syringic acid.

## 2. Materials and Methods

### 2.1. Materials and Reagents

Syringic acid (≥95%) was acquired from Sigma-Aldrich (St. Louis, MO, USA). Trimesic acid (benzene-1,3,5-tricarboxylic acid, ≥98%), ferrous chloride tetrahydrate (FeCl_2_·4H_2_O), sodium hydroxide (NaOH), and ethanol (95%) were procured from standard commercial suppliers. Ultrapure water was generated using a Milli-Q system. All reagents were used as received without further purification.

### 2.2. Animals

Healthy male Sprague Dawley (SD) rats (6–8 weeks old; 200–250 g) were procured from the Laboratory Animal Facility of the Department of Science and Technology–Industrial Technology Development Institute (DOST–ITDI, Taguig City, Philippines). The animals were housed under controlled environmental conditions, maintaining a temperature of 25 ± 2 °C, a relative humidity of 45 ± 5%, and a 12-h light/dark cycle. Rats were provided with standard laboratory chow and distilled water ad libitum and acclimatized for seven days before the study in 20 × 18 × 7-inch plastic cages with metal cover cages with beddings. Healthy test animals were used during the experimentation upon examination by the licensed veterinarian. Test animals were allocated to the respective treatment groups via blind selection to ensure randomization. Potential confounders were minimized through randomization and environmental standardization. As previously described, animals were allocated to treatment groups using simple random sampling (lottery method). To control for environmental variables, cages from different groups were interspersed across the rack shelves rather than clustered by group. Furthermore, dosing and blood sampling procedures were performed in an alternating order between groups to ensure that no specific group was influenced by time-of-day effects or circadian variations in metabolism. The licensed veterinarian and assistants were aware of this during the different stages of the experiments.

The Sprague Dawley (SD) rat was selected for this pharmacokinetic analysis given its extensive validation as a standard model for toxicological and bioavailability evaluations. Its physiological size permits serial blood sampling from individual subjects, which is critical for generating precise, longitudinal pharmacokinetic profiles and minimizing inter-subject variability without the need for composite sampling required in smaller species. Furthermore, the rat model offers significant relevance to human biology, particularly regarding gastrointestinal absorption mechanisms and hepatic metabolic pathways (Phase I and II), making it a predictive surrogate for evaluating oral drug delivery systems. By challenging the MIL-100(Fe)-10% formulation against the rat’s robust metabolic system, the study can effectively validate the carrier’s ability to protect syringic acid from first-pass metabolism and facilitate sustained release, providing proof-of-concept data that is translatable to potential human applications.

Experimental protocols were reviewed and approved by the Institutional Animal Care and Use Committee (IACUC) of DOST–ITDI, in accordance with Bureau of Animal Industry (BAI) guidelines (Protocol No. AR-2024-0134; approved 4 March 2024). All procedures were performed under the supervision of a licensed veterinarian following a 12-h fasting period with water provided ad libitum.

### 2.3. Ethics and Study Design

Prior to the commencement of the study, a comprehensive protocol detailing the research question, experimental design, and analysis plan was submitted to the DOST–ITDI Institutional Animal Care and Use Committee (IACUC) for approval, in strict compliance with Bureau of Animal Industry (BAI) guidelines. The study followed this approved protocol (Approval No. AR-2024-0134; approved 4 March 2024). The protocol was not registered in a public database.

### 2.4. Methods

#### 2.4.1. Synthesis of MIL-100-XX%

MIL-100(Fe)-XX% was synthesized following the procedure previously described by Luo and co-workers [11] and as reported by our previous journal article [8]. The study employed the modification in the use of syringic acid as a substitute for trimesic acid in varying ratios. The corresponding label of the name was used for every percentage of syringic acid as the organic linker used, which is labeled as “MIL-100(Fe)-XX%”. The synthesis was performed by dissolving iron(II) chloride tetrahydrate (1.14 mmol, 226.64 mg), the organic linker (0.79 mmol), and sodium hydroxide (2.28 mmol, 91.2 mg) in 60 mL of deionized water. The specific mixed-linker ratios utilized are detailed in Table 1. The mixture was stirred at room temperature for 24 h. The resulting modified MIL-100(Fe) precipitate was collected by centrifugation (5000 rpm, 10 min) and subsequently washed. The washing process involved three cycles of resuspension in 60 mL of deionized water at [temperature]°C for 30 min, followed by centrifugation [3]. The washing of the modified MIL-100(Fe) was performed with 95% ethanol under the same conditions as those of the deionized water washing. The separated particles were dried under vacuum at 80 °C for 30 min.

#### 2.4.2. Post-Synthetic Confirmation

The crystallinity of the synthesized MIL-100(Fe)-XX% was verified using powder X-ray diffraction (PXRD) by comparing the observed Bragg peaks with the calculated diffractogram of MIL-100(Fe). Analysis was conducted on a Bruker D8 Phase diffractometer (Bruker, Taiwan) using monochromated Cu Kα radiation (30 kV, 10 mA). Data were collected in the 2θ range of 2° to 50°, with a step size of 0.03° and an integration time of 0.5–3.5 s per step [12,13]. This low-angle range was selected to capture the characteristic reflections of the mesoporous MIL-100(Fe) framework located between 2° and 4°.

#### 2.4.3. Syringic Acid Impregnation and Quantification

Following the synthesis of the mixed-linker framework (where syringic acid acts as a structural co-ligand), a post-synthetic impregnation step was employed to maximize the drug payload. While the initial substitution of syringic acid serves to engineer the pore topology (expanding pore width), the stoichiometric limit of this substitution (~10%) is insufficient for therapeutic dosing. Therefore, the bulk of the therapeutic payload was introduced via physical adsorption into the expanded pores of the MIL-100(Fe)-10% matrix. Syringic acid was loaded into the carrier via a simple drug impregnation method, following established protocols reported by Cunha et al. and Singco et al. [14,15].

Briefly, 100 mg of MIL-100(Fe)-XX% was suspended in an ethanolic solution of syringic acid at a 1:1 weight ratio (drug-to-carrier), using a solvent volume of 100 µL per mg of carrier. The mixture was stirred at 75 rpm for varying durations: 12, 24, 36, and 48 h. Following incubation, the suspension was centrifuged at 10,000 rpm for 10 min, and the supernatant was collected for subsequent analysis. To eliminate loosely bound drug molecules, the SYA@MIL-100(Fe)-XX% particles were subjected to three washing cycles using 12 mL of 95% ethanol. Each cycle included centrifugation at 10,000 rpm for 10 min. The purified product was subsequently dried at 80 °C and stored for analysis [14]. To investigate the influence of drug concentration on loading capacity, the MIL-100(Fe)-XX% to syringic acid ratio was increased to 1:2 (*w*/*w*). This modification allowed for an assessment of whether a higher concentration of syringic acid would enhance the total amount of drug encapsulated within the framework.

The amount of syringic acid impregnated into MIL-100(Fe)-XX% was calculated indirectly by quantifying the unabsorbed drug in the supernatant. Analysis was performed using a Shimadzu 2050C High-Performance Liquid Chromatography (HPLC) system (Shimadzu, Kyoto, Japan) equipped with a photodiode array (PDA) detector. Separation was achieved using an InertSustain C18 column (5 µm, 150 × 4.6 mm; GL Sciences, Tokyo, Japan). The mobile phase consisted of water/methanol (70:30 *v*/*v*) containing 0.1% acetic acid, delivered at a flow rate of 1.0 mL/min with a detection wavelength of 272 nm [14,16]. Free syringic acid present during the synthesis of MIL-100(Fe)-XX% was quantified using the same HPLC method. This value was incorporated into the mass balance to account for both the total syringic acid content and the specific amount successfully impregnated into the framework.

(1) $$Drug\;Loading\;Percent=\frac{\left(TS-SS\right)+FS}{M}\times 100$$

where *TS*—total amount of syringic acid (mg) used in the loading protocol; *SS*—amount of syringic acid in supernatant (mg) in the loading protocol; *FS*—amount of syringic acid detected in the supernatant (mg) in the synthesis protocol; *M*—amount of MIL-100(Fe)-XX% used (mg).

#### 2.4.4. Characterization of SYA@MIL-100(Fe)-XX%

The formulation demonstrating the highest drug-loading capacity among the 1:1 and 1:2 ratios was selected for comprehensive characterization.

##### Nitrogen Sorption Isotherms

Nitrogen adsorption–desorption isotherms were acquired at 77.35 K using a Micromeritics ASAP 2020 system (Micromeritics, Taipei, Taiwan). Prior to measurement, the MIL-100(Fe)-XX% samples were degassed under high vacuum; this involved heating at 90 °C for 1 h, followed by a ramp (10 °C min^−1^) to 350 °C for 6 h to remove residual solvents. The Brunauer–Emmett–Teller (BET) specific surface area (SBET, m^2^ g^−1^) was calculated from the linear region of the isotherm (P/P_0_ = 0.05–0.30), while the total pore volume (V_total_, cm^3^ g^−1^) was determined from nitrogen uptake at a relative pressure (P/P_0_) of approximately 0.99 [17].

##### Thermogravimetric Analysis (TGA)

Thermogravimetric analysis (TGA) was employed to assess the thermal stability of both MIL-100(Fe)-XX% and SYA@MIL-100(Fe)-XX%. Samples (5–10 mg) were placed in ceramic pans and subjected to a temperature ramp from 50 to 800 °C at a rate of 10 °C min^−1^ under a nitrogen flow of 20 mL min^−1^. The resulting thermograms (weight percentage vs. temperature) were analyzed to elucidate thermal degradation profiles and evaluate the effect of syringic acid encapsulation on framework stability [18].

##### Powder X-Ray Diffraction (PXRD)

The crystallinity of the SYA@MIL-100(Fe)-10% was confirmed using PXRD, following the same parameters described above for the MIL-100(Fe)-10% (Section 2.4.2: Post Synthetic Confirmation).

##### Fourier-Transform Infrared Spectroscopy (FTIR)

Fourier-transform infrared (FTIR) spectroscopy was employed to characterize surface functional groups and verify the successful encapsulation of syringic acid. Samples were prepared via the potassium bromide (KBr) pellet method and analyzed across the 400–4000 cm^−1^ spectral range. The obtained spectra were examined for characteristic vibrational shifts and drug-specific bands to confirm the integrity of the SYA@MIL-100(Fe) system.

##### Scanning Electron Microscope (SEM)

Surface morphology was visualized using field emission scanning electron microscopy (FE-SEM) on a JEOL JEM-700F system (Hsinchu, Taiwan). Prior to imaging, samples were vacuum-dried and mounted onto stubs using double-sided carbon tape. To prevent charging and enhance conductivity, the specimens were sputter-coated with platinum under a low-pressure argon atmosphere. Micrographs were acquired at an accelerating voltage of 15 kV and 20,000× magnification to assess particle geometry and size distribution [16,19].

##### Particle Size Determination

The average hydrodynamic diameter and particle size distribution were determined via dynamic light scattering (DLS) using a Horiba Nanopartica SZ-100V2 analyzer (Horiba Scientific, Kyoto, Japan). To facilitate deagglomeration and ensure homogeneity, samples were suspended in ultrapure water (100 ppm) and subjected to ultrasonic dispersion (40 kHz) for 10 min. Measurements were conducted at 25 °C, utilizing standard optical parameters for the aqueous medium (refractive index: 1.33; dielectric constant: 78.3) [16,19].

#### 2.4.5. In Vitro Drug Release Study

The in vitro dissolution behavior of pure syringic acid and the SYA@MIL-100(Fe)-XX% nanocarrier was investigated using the sample-and-separate technique. To mimic various physiological environments, the study utilized four distinct media: ultrapure water, simulated gastric fluid (0.1 N HCl, pH 2.0), simulated intestinal fluid (PBS, pH 6.8), and simulated systemic circulation (PBS, pH 7.4). The experiments were conducted in 100 mL of medium maintained at 37.5 ± 0.5 °C with constant agitation (75 rpm). Sink conditions were strictly upheld by ensuring the drug concentration remained below 33% of its saturation solubility. At predetermined intervals ranging from 5 to 1800 min, 1000 µL aliquots were collected and immediately replaced with fresh pre-warmed medium. The collected samples were centrifuged (5000 rpm, 10 min) to separate the matrix; the supernatant was analyzed via HPLC, while the sedimented pellets were redispersed into the release vessel. To characterize the release mechanism, data were fitted to five kinetic models (Zero-order, First-order, Higuchi, Korsmeyer–Peppas, and Hixson–Crowell). The model yielding the highest correlation coefficient (r^2^) was identified as the best fit [20].

#### 2.4.6. Acute Oral Toxicity

The safety profile was assessed in accordance with the OECD 423 Acute Toxic Class Method. Healthy male Sprague Dawley rats weighing between 200 and 250 g served as the test subjects. Prior to administration, a 1 mL blood sample was collected via the tail vein to determine baseline hepatic and renal function.

The acute oral toxicity profile of the formulation was evaluated according to OECD Guideline 423 to establish the safety margin and determine the appropriate hazard classification. This method was chosen because it provides a reproducible and scientifically valid assessment of acute toxicity without the need for precise LD_50_ determination, which is often unnecessary for risk assessment. Furthermore, OECD 423 allows for the use of a limit test (typically at 2000 mg kg^−1^), which is particularly appropriate for testing drug delivery systems and excipients like MIL-100(Fe) that are expected to have low toxicity profiles. The results derived from this method are internationally accepted for regulatory purposes and GHS classification.

Hepatic and renal function were monitored by quantifying alanine aminotransferase (ALT), aspartate aminotransferase (AST), blood urea nitrogen (BUN), and creatinine levels. Adhering to the limit test protocol, an initial cohort of three rats received a dose of 2000 mg kg^−1^. Upon confirming the absence of morbidity or mortality after a one-week observation, a confirmatory group of three additional rats was administered the same dose, bringing the total sample size to six. A separate control group was not utilized; instead, the study employed a self-controlled design where Day 14 biochemical parameters were compared against each animal’s pre-dose baseline [21]. The primary outcome measured is the survival of the test animal after 14 days of observation.

Clinical observations for toxicological symptoms were conducted over a 14-day post-dosing period. At the study’s conclusion (Day 14), animals were anesthetized using Zoletil 50 (50 mg kg^−1^; composed of 50 mg tiletamine, 50 mg zolazepam, and 57.7 mg mannitol mL^−1^) to facilitate blood collection via cardiac puncture for final biochemical profiling. Subsequently, the subjects were euthanized via carbon dioxide inhalation, and the liver and kidneys were excised to evaluate potential histopathological alterations.

#### 2.4.7. Oral Bioavailability and Tissue Distribution

Based on methods established by Ding et al., Sun et al., and Santos et al., the study quantified the bioavailability of syringic acid and its distribution in the liver and kidneys [16,22,23]. The primary rationale for conducting this bioavailability study is to overcome the pharmacokinetic limitations of free syringic acid. The primary outcome is the determination of the area under the curve (AUC) of the syringic acid and SYA@MIL-100(Fe)-10%. Sprague Dawley rats were divided into 10 groups (n = 3 per time point) corresponding to sampling intervals of 15, 30, 60, 120, 240, 480, 1080, 1440, 2880, and 4320 min. The number of test animals was based on the lowest allowable number, in compliance with the principle of 3R, which is still statistically valid. For oral administration, rats received either pure syringic acid (as the control group) or an equivalent dose of SYA@MIL-100(Fe)-XX% at 100 mg kg^−1^. For intraperitoneal (i.p.) administration, doses were adjusted to 25 mg kg^−1^ for pure syringic acid and 1.99 mg kg^−1^ for the loaded framework to prevent abdominal distress and ensure animal welfare. All subjects were fasted prior to administration.

##### Collection and Processing of Blood Samples

At predetermined time intervals, animals were anesthetized using Zoletil 50 (1 mg kg^−1^) to facilitate blood withdrawal via intracardiac aspiration. Following coagulation, the samples were centrifuged at 4000 rpm (5 min, 4 °C) to separate the serum. Deproteinization was achieved by mixing the serum with an equal volume of cold methanol, followed by centrifugation to recover the supernatant. This precipitation step was reiterated until the supernatant remained clear, indicating complete protein removal. The resulting solution was then dried under vacuum and stored for HPLC quantification.

##### Collection and Processing of Organ Samples

Post-mortem, hepatic and renal tissues were excised, blotted to remove excess moisture, and weighed. Homogenization was performed in Normal Saline Solution (NSS) using a ratio of 1 mL solvent per 700 mg of tissue. To extract the target compound and precipitate cellular debris, the homogenates were mixed with an equivalent volume of absolute ethanol and centrifuged (5000 rpm, 10 min). A similar deproteinization protocol was applied to serum samples using absolute ethanol, repeated until the supernatant remained clear. The organic solvent was subsequently removed via vacuum evaporation prior to analysis.

##### Syringic Acid Quantification

Chromatographic separation was achieved using a mobile phase of water (0.1% acetic acid) and methanol. The HPLC system operated with a linear gradient elution at a flow rate of 1.0 mL min^−1^. The method employed a gradient program starting at 80:20 (water/methanol *v*/*v*), ramping linearly to 40:60 over 10 min. The column temperature was held at 30 °C, and absorbance was monitored at 218 nm via a photodiode array (PDA) detector.

#### 2.4.8. Statistical Analysis

Results are expressed as the mean ± standard deviation (SD) derived from three independent replicates. Data analysis was performed using the SPSS 20.0 software package (SPSS Inc., Chicago, IL, USA). To evaluate differences within the same group, paired-sample t-tests were utilized, whereas variations across multiple groups were assessed via one-way analysis of variance (ANOVA). Subsequent pairwise comparisons were conducted using Tukey’s Honestly Significant Difference (HSD) post hoc test. Statistical significance was established at a confidence level of 95% (*p* < 0.05).

## 3. Results

### 3.1. Synthesis of MIL-100(Fe)-XX%

The synthesized MIL-100(Fe)-XX%s (Figure S1) were prepared by increasing the ratio of the syringic acid as the organic linker in relation to trimesic acid, such as 10%, 20%, 30%, 40%, 50%, and 100%. It can be noted that the color of the different MIL-100(Fe)-XX% displayed a change from brown to dark bluish-brown color during the 24-h synthesis, following the method as described by Luo and co-workers. It can be noted that the difference in the color from the MIL-100(Fe) (Figure S2A), as synthesized in the previously reported journal, and MIL-100(Fe)-10% (Figure S2B) was due to the presence of syringic acid. The former was noted to be reddish-brown (rusty) in color, while the latter was described as darker reddish-brown in color.

**Figure 3:**
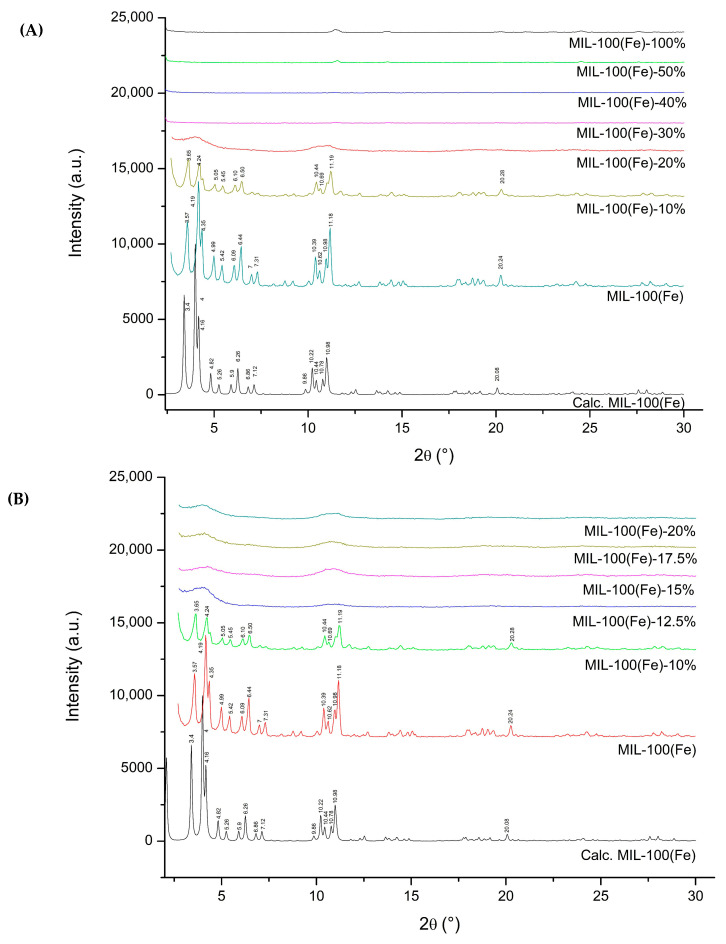
PXRD plot of (**A**) varying percentage amount of syringic acid as organic linker (10%, 20%, 30%, 40%, 50%, and 100%) and (**B**) varying percentage amount of syringic acid as organic linker (10%, 12.5%, 15%, 17.5%, and 20%).

Powder X-ray diffraction (PXRD) was utilized to verify the structural stability of the MIL-100(Fe) framework following drug loading, a critical quality attribute of metal–organic frameworks (MOFs). As shown in Figure 3A, the characteristic Bragg reflections observed in the simulated MIL-100(Fe) pattern—specifically at 2θ values of 3.40, 4.00, 4.16, 4.82, 5.26, 5.92, 6.82, 7.14, 10.22, 10.44, 10.78, 10.98, and 20.08—were preserved in both the unmodified carrier and the formulation containing 10% syringic acid. However, these peaks were notably absent in samples with drug loadings of 20% or higher.

Detailed analysis of the 10–20% loading range (Figure 3B) identified the tipping point for framework collapse. At 10% loading, peaks were clearly resolved at 3.65, 4.24, 5.05, 5.45, 6.10, 6.50, 10.44, 10.69, 11.19, and 20.28. However, increasing the load to 12.5% resulted in the disappearance of these reflections, a trend that continued at 15%, 17.5%, and 20%, indicating that the carrier cannot support drug levels above 10% without undergoing structural degradation. The observed diffraction patterns are in excellent agreement with both the theoretical simulation and the existing literature for MIL-100(Fe) [4].

With the supporting data from the PXRD, MIL-100(Fe)-10% was used for further characterization and testing throughout this article.

### 3.2. Syringic Acid Impregnation and Quantification

**Figure 4:**
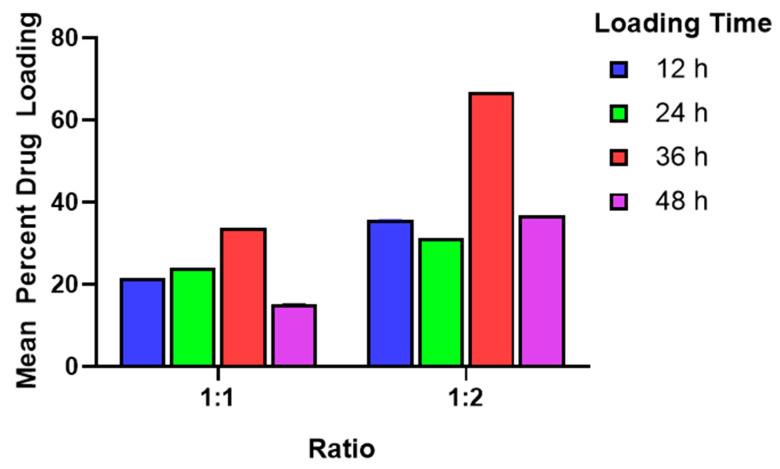
Average encapsulation efficiency of syringic acid assessed across four distinct incubation intervals. Data presented is mean ± S.E.M.

The encapsulation of syringic acid into the MIL-100(Fe)-10% framework was optimized by varying the incubation time (12, 24, 36, and 48 h) and the carrier-to-drug weight ratio (1:1 and 1:2). As illustrated in Figure 4, these parameters significantly influenced loading efficiency. For the 1:1 formulation, the drug uptake did not follow a linear progression with time. Instead, the highest loading was achieved at 36 h (33.70% ± 0.00%), followed by 24 h (24.05% ± 0.01%) and 12 h (21.52% ± 0.01%). Interestingly, extending the incubation to 48 h resulted in the lowest loading capacity (15.20% ± 0.01%). The loading time at 36 h resulted in a significantly greater mean drug loading compared to the other loading times at a *p*-value of less than 0.001. An increasing trend in the mean percent drug loading has been noted for the 1:2 ratio, namely, 66.85% ± 0.004% (36 h) < 36.67% ± 0.01% (48 h) < 35.73% ± 0.01% (12 h) < 31.31% ± 0.01% (24 h). It is noteworthy that the mean percent drug loading at 36 h resulted in a significantly greater mean drug loading compared to the other loading points at a *p*-value of less than 0.001. In both ratios, the 36-hour loading time resulted in the highest drug loading, suggesting a potential optimal duration for syringic acid incorporation into MIL-100(Fe). The significant decrease in mean drug loading observed at 48 h may be attributed to partial framework degradation or structural collapse resulting from prolonged exposure to syringic acid. This suggests that the chemical environment or the acidity of the guest molecule may compromise the long-term stability of the MIL-100(Fe) matrix.

Two-way ANOVA demonstrated that both the loading duration and the MIL-100(Fe)-10%-to-syringic-acid ratio, as well as their interaction, exerted a statistically significant influence on the mean drug-loading capacity (*p* < 0.001). These findings demonstrate that encapsulation efficiency is governed by the complex interplay between drug concentration and incubation duration.

### 3.3. PXRD

**Figure 5:**
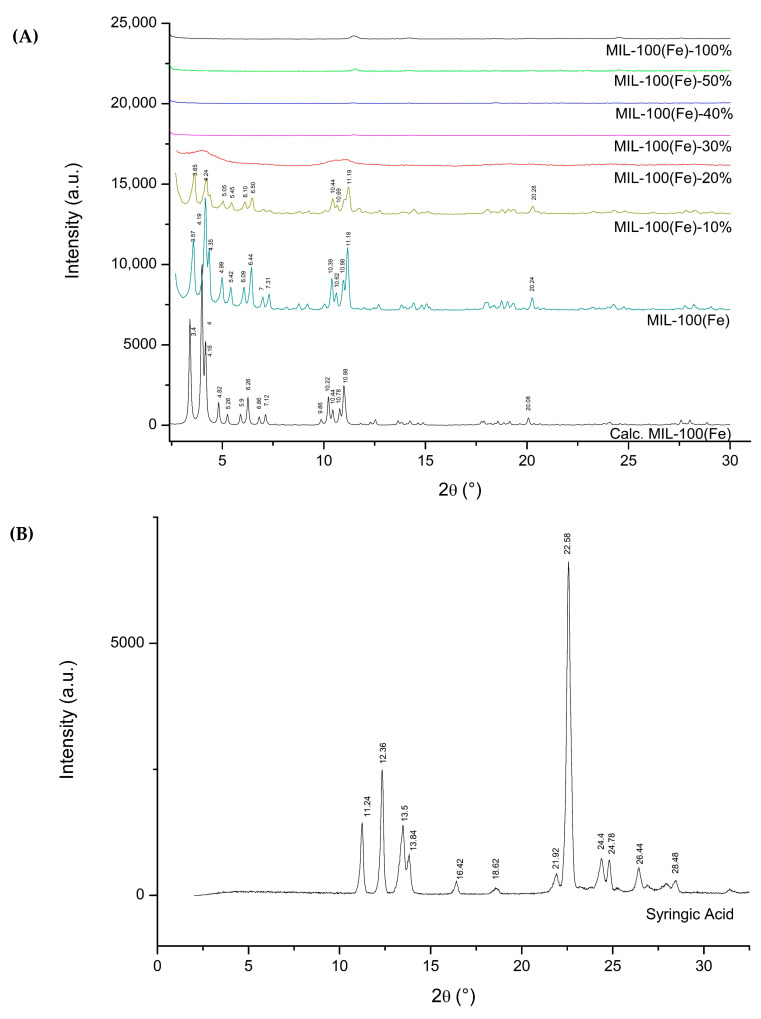
PXRD plot of (**A**) MIL-100(Fe)-10% loaded with syringic acid at different time points (12-, 24-, 36-, and 48-hours) and (**B**) syringic acid.

PXRD analysis of the synthesized MIL-100(Fe)-10% confirmed that the fundamental crystalline topology of the framework was preserved following the introduction of syringic acid, with no evidence of phase impurities or amorphous degradation at this loading level. Furthermore, post-loading PXRD was employed to assess the structural integrity of the framework following exposure to syringic acid across all time intervals (12–48 h) and mass ratios (1:1 and 1:2). The observed Bragg peaks aligned closely with previously reported patterns and the simulated crystalline structure of MIL-100(Fe) [20]. Although minor deviations in peak positions were noted (Figure 5A), the overall retention of characteristic reflections indicates that the long-range crystalline order was largely preserved during the drug-loading process.

Notably, the characteristic Bragg peaks of MIL-100(Fe)-10% were preserved across all loading durations (12–48 h), as shown in Figure 5B. This retention of the diffraction profile suggests that the framework maintains its structural stability and crystallinity even upon a secondary exposure to syringic acid. Furthermore, the absence of new diffraction peaks corresponding to crystalline syringic acid—when compared to the pure drug pattern in Figure 5A—indicates that the drug did not recrystallize or remain as a free phase on the carrier surface. The lack of external drug reflections strongly supports the conclusion that syringic acid was successfully encapsulated within the pores of the MIL-100(Fe)-10% matrix rather than simply adsorbed externally. These findings are consistent with previous reports on the robust nature of modified MIL-100(Fe) systems, which retain high porosity and structural integrity during the host–guest encapsulation of active molecules.

### 3.4. Fourier-Transform Infrared Spectroscopy

**Figure 6:**
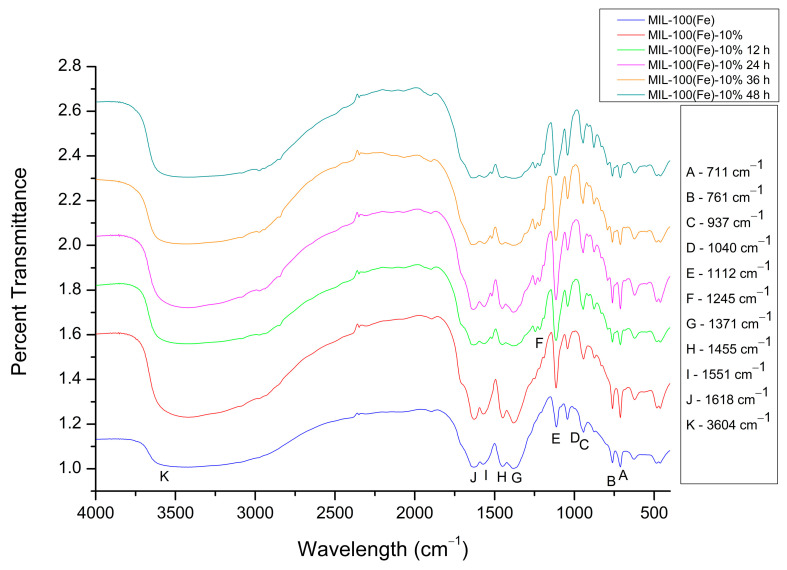
FTIR spectra of MIL-100(Fe), MIL-100(Fe)-10%, and different preparations (SYA@MIL-100(Fe)-10% XXhrs).

Fourier-transform infrared (FTIR) spectroscopy was employed to characterize the functional groups of MIL-100(Fe), the modified MIL-100(Fe)-10%, and the drug-loaded SYA@MIL-100(Fe)-10% formulations (Figure 6). This analysis served to verify the chemical integrity of the framework post-modification and to confirm the successful incorporation of syringic acid by monitoring characteristic vibrational modes. The characteristic spectral peaks of MIL-100(Fe) were largely conserved following the mixed-linker modification (MIL-100(Fe)-10%) and the subsequent impregnation of syringic acid (SYA@MIL-100(Fe)-10%), as shown in Figure 6. Specifically, the vibrational modes of the trimesic acid linker remained prominent: the O–H stretching from hydrogen-bonded dimers at 3604 cm^−1^ (Peak K), C=C aromatic stretching at 1618 cm^−1^ (Peak J), and carboxylate C–O stretching at 1551 cm^−1^ (Peak I). Other conserved reflections included aromatic C–C stretching at 1455 cm^−1^ (Peak H), C–H rocking at 1371 cm^−1^ (Peak G), and C–O stretching at 1112 cm^−1^ (Peak E). Furthermore, O–H bending modes were observed at 1040 and 937 cm^−1^ (Peaks D and C), alongside C–H out-of-plane bending at 761 and 711 cm^−1^ (Peaks B and A). The lack of dramatic spectral shifts is attributed to the structural similarities between the trimesic acid (Figure 1C) and syringic acid (Figure 1B) frameworks, as both molecules possess analogous phenolic and carboxylic functional groups.

In congruence with our previously reported article, a distinct peak is detected at 1245 cm^−1^ (Peak F), corresponding to the C-O stretching vibration of an aromatic ether, as strongly observed in the SYA@MIL-100(Fe)-10% XX hrs. Despite being present as a part of the mixed-linker approach, the signal in the MIL-100(Fe)-10% was not as prominent compared to the SYA@MIL-100(Fe)-10% XX hrs. Possible reasons include the deprotonation of the carboxylic acid in syringic acid, and the formation can potentially lead to changes in the infrared spectra behavior of the methoxy group, as reported in the conversion of vanillin to its oxyanionic form [24]. In another study, when an organic linker is used in the formation of a metal organic framework, the methoxy substituent, a strongly electron-donating group, interacts with a more delocalized pi-system extending toward the secondary building unit (SBU) of MIL-1000(Fe), which is Fe_2_O_3_. The delocalization resulted in a change of the dipole moment with the aryl methoxy stretching vibration, which resulted in a weaker infrared band [25]. The presence of the Peak F indicates the presence of syringic acid in the SYA@MIL-100(Fe)-XX hrs.

### 3.5. Nitrogen Adsorption–Desorption

**Figure 7:**
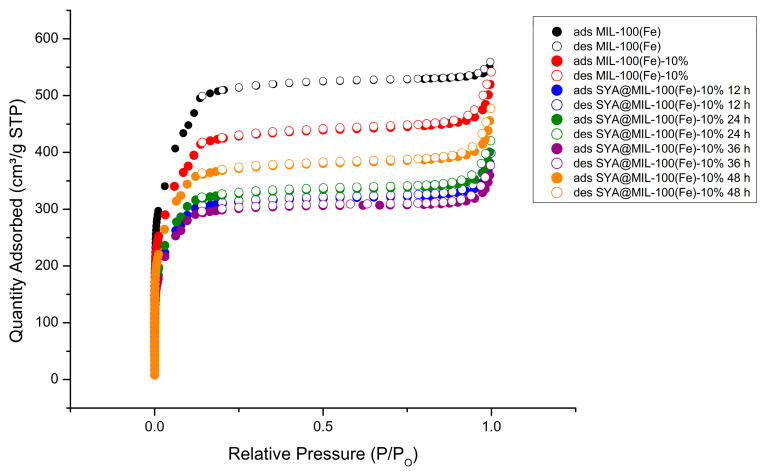
BET isotherm plot of MIL-100(Fe), MIL-100(Fe)-10%, and different preparations (SYA@MIL-100(Fe)-10% XXhrs).

The BET isotherm plot was plotted as relative pressure (P/P_o_) versus Quantity Adsorbed (cm^3^/ g STP) (Figure 7). Table 2 summarizes the textural characteristics of the MIL-100(Fe), MIL-100(Fe)-10%, and SYA@MIL-100(Fe)-10% XX hrs, such as BET surface area and total pore volume. The BET surface of MIL-100(Fe) was recorded at 2028.35 m^2^ g^−1^, but modification using the mixed-linker approach led to a lower BET surface area for MIL-100(Fe)-10% at 1684.36 m^2^ g^−1^, accounting for a 16.96% reduction. In congruence with our previous article [2], reductions in the BET surfaces were noted after syringic acid impregnation at different time points, such as 1237.67 m^2^ g^−1^ (12 h), 1294.45 m^2^ g^−1^ (24 h), 1194.49 m^2^ g^−1^ (36 h), and 1466.20 m^2^ g^−1^ (48 h) with corresponding reductions based on the BET surface area of MIL-100(Fe)-10% as 26.52%, 23.15%, 29.08%, and 12.95%, respectively. This decrease indicates the successful impregnation of syringic acid into the internal surface or blocking of the pore opening as depicted in Figure 7.

Other changes in textural characteristics due to the use of the mixed-linker approach include a 6.69% decrease in the total pore volume (0.855916 cm^3^ g^−1^ (MIL-100(Fe) and 0.798689 cm^3^ g^−1^ (MIL-100(Fe)-10%), a 9.90% decrease in the micropore volume (0.297397 cm^3^ g^−1^ (MIL-100(Fe) and 0.267967 cm^3^ g^−1^ (MIL-100(Fe)-10%), a 4.98% decrease in the mesopore volume (0.558519 cm^3^ g^−1^ (MIL-100(Fe) and 0.530722 cm^3^ g^−1^ (MIL-100(Fe)-10%), and a 67.03% increase in the pore width (29.717 Å (MIL-100(Fe) and 49.636 Å (MIL-100(Fe)-10%).

With respect to the textural characteristics of MIL-100(Fe)-10%, changes were noted post-impregnation of syringic acid at the different time points. Reductions in the total pore volume after syringic acid impregnation were noted to be 0.591158 cm^3^ g^−1^ (25.99%), 0.62355 cm^3^ g^−1^ (21.93%), 0.56121 cm^3^ g^−1^ (29.73%), and 0.707342 cm^3^ g^−1^ (11.44%) for 12, 24, 36, and 48 h, respectively. On the contrary, increases in the micropore volume after syringic acid impregnation were noted to be 0.340373 cm^3^ g^−1^ (27.02%), 0.366032 cm^3^ g^−1^ (36.60%), 0.336622 cm^3^ g^−1^ (25.62%), and 0.407326 cm^3^ g^−1^ (52.01%) for 12, 24, 36, and 48 h, respectively. The same changes as in the total pore volume were noted with the mesopore volume as 0.250785 cm^3^ g^−1^ (52.75% reduction), 0.25751 cm^3^ g^−1^ (51.48% reduction), 0.224588 cm^3^ g^−1^ (57.68% reduction), and 0.300016 cm^3^ g^−1^ (43.47% reduction) for 12, 24, 36, and 48 h, respectively. Lastly, increases in pore width were noted to be 60.229 Å (21.34%), 59.689 Å (20.25%), 58.936 Å (18.74%), and 58.178 Å (17.21%) for 12, 24, 36, and 48 h, respectively.

### 3.6. Thermogravimetric Analysis

**Figure 8:**
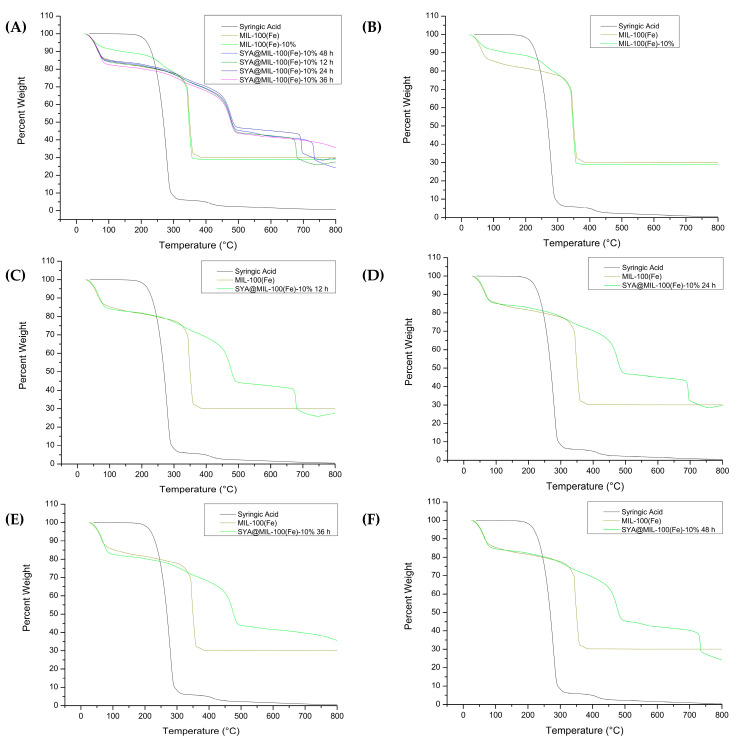
Thermograms of (**A**) syringic acid, MIL-100(Fe), MIL-100(Fe)-10%, and MIL-100(Fe) XX h; (**B**) syringic acid, MIL-100(Fe), and MIL-100(Fe)-10%; (**C**) syringic acid, MIL-100(Fe)-10%, and SYA@MIL-100(Fe)-10% at 12 h; (**D**) syringic acid, MIL-100(Fe)-10%, and SYA@MIL-100(Fe)-10% at 24 h; (**E**) syringic acid, MIL-100(Fe)-10%, and SYA@MIL-100(Fe)-10% at 36 h; and (**F**) syringic acid, MIL-100(Fe)-10%, and SYA@MIL-100(Fe)-10% at 48 h.

The thermograms of syringic acid with MIL-100(Fe), MIL-100(Fe)-10%, and SYA@MIL-100(Fe)-10%—XX hrs are illustrated in Figure 8A. Specifically, comparative thermograms containing syringic acid, MIL-100(Fe), and MIL-100(Fe)-10% are presented in Figure 8B, while those of syringic acid, MIL100(Fe)-10%, and MIL100(Fe)-10% XX hrs are shown in Figure 8C–F. Residual weights were noted at 30.08% (MIL-100(Fe)), 28.84% (MIL-100(Fe)-10%), 27.51% (SYA@MIL-100(Fe)-10% at 12 h), 29.77% (SYA@MIL-100(Fe)-10% at 24 h), 35.67 (SYA@MIL-100(Fe)-10% at 36 h), and 24.43% (SYA@MIL-100(Fe)-10% at 48 h).

### 3.7. Surface Morphology and Particle Size Analysis

**Figure 9:**
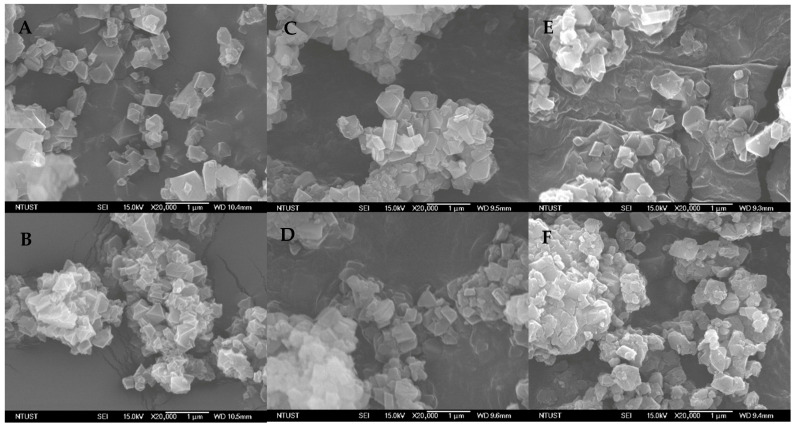
Micropictograph of (**A**) MIL-100(Fe); (**B**) MIL-100(Fe)-10%; (**C**) SYA@MIL-100(Fe)-10% at 12 h; (**D**) SYA@MIL-100(Fe)-10% at 24 h; (**E**) SYA@MIL-100(Fe)-10% at 36 h; and (**F**) SYA@MIL-100(Fe)-10% at 48 h taken at 10 kV and 20,000× magnification.

The surface morphology of the synthesized frameworks was examined via FE-SEM at 15 kV and 20,000× magnification (Figure 9A–F). Despite the implementation of the mixed-linker approach, the crystalline habit of the particles remained unaltered, which is in high congruence with the PXRD findings. Specifically, MIL-100(Fe)-10% (Figure 9B) retained the characteristic triangular-based pyramidal (octahedral) morphology observed in the parent MIL-100(Fe) (Figure 9A). This specific geometry is advantageous for drug delivery, as it supports a high surface-area-to-volume ratio, thereby facilitating efficient drug impregnation and potentially enhancing therapeutic efficacy. Furthermore, the morphology remained stable following prolonged exposure to syringic acid across all time intervals. These observations align with our post-impregnation PXRD data and correspond to our previously reported findings on the structural resilience of the MIL-100(Fe) matrix.

The mean hydrodynamic particle size obtained using dynamic light scattering of MIL-100(Fe)-10% (360.63 ± 10.77 nm) was significantly larger compared to SYA@MIL-100(Fe)-10% XX hrs, specifically at 177.07 ± 7.26 nm (12 h), 142.23 ± 7.96 nm (24 h), 210.70 ± 1.23 nm (36 h), and 276.27 ± 5.52 nm (48 h) at *p*-values less than 0.001. The significant decrease in the particle size might be attributed to the initial aggregation of MIL-100(Fe)-10% after synthesis, activation, drying, and storage.

### 3.8. In Vitro Drug Release

**Figure 10:**
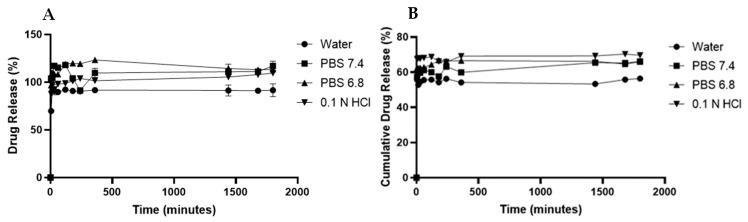
In vitro dissolution profile of (**A**) syringic acid and (**B**) SYA@MIL-100(Fe)-10% evaluated in four distinct solvents: water (•). HCl—hydrochloric acid (▼); PBS—phosphate-buffered saline at pH 7.4 (▪) and 6.8 (▲). Data presented is mean ± S.E.M.

Figure 10 illustrates the in vitro dissolution profiles of syringic acid in four distinct media: water, PBS (pH 7.4 and pH 6.8), and 0.1 N HCl. Over a 30-hour period, the modified MIL-100(Fe) carrier demonstrated a sustained-release behavior. The cumulative release was highest in acidic conditions (0.1 N HCl: 68.23% ± 0.42%), followed by PBS pH 6.8 (64.65% ± 0.55%), PBS pH 7.4 (61.52% ± 0.93%), and finally water (54.97% ± 0.37%). These results indicate a controlled and prolonged release rate within simulated physiological fluids. To characterize the release mechanism, the experimental data were fitted to five standard kinetic models: zero-order, first-order, Higuchi, Korsmeyer–Peppas, and Hixson–Crowell.

### 3.9. Acute Oral Toxicity

In adherence to OECD 423 guidelines, the acute oral safety profile of both pure syringic acid and the SYA@MIL-100(Fe)-10% formulation was investigated. Exposure to a limit dose of 2000 mg kg^−1^ caused no lethality throughout the 14-day monitoring phase. The absence of mortality suggests that both the drug and the loaded carrier exhibit a favorable safety profile at this concentration. No data from the test animal was excluded from the data analysis and presentation. No significant adverse effects were noted after the administration of the test compound. Serological analyses demonstrated a significant reduction in serum glutamic pyruvic transaminase (SGPT) levels following administration of syringic acid (from 87.96 ± 14.9 to 46.73 ± 3.66 U L^−1^, *p* = 0.02, n = 6) and SYA@MIL-100(Fe)-10% (from 70.59 ± 4.93 to 41.41 ± 2.39 U L^−1^, *p* = 0.002, n = 6). Similarly, serum glutamic oxaloacetic transaminase (SGOT) levels significantly decreased in both treatment groups (syringic acid: from 337.30 ± 34.87 to 200.63 ± 19.17 U L^−1^, *p* = 0.01, n = 6; SYA@MIL-100(Fe)-10%: from 283.74 ± 36.08 to 149.02 ± 14.88 U L^−1^, *p* = 0.016, n = 6). No significant changes were observed in blood urea nitrogen (BUN) levels after treatment (syringic acid: from 14.71 ± 0.51 to 15.57 ± 1.59 mg dL^−1^, *p* = 0.548, n = 6; SYA@MIL-100(Fe)-10%: from 15.17 ± 0.67 to 15.89 ± 1.77 mg dL^−1^, *p* = 0.724, n = 6), nor in serum creatinine concentrations (syringic acid: from 0.58 ± 0.01 to 0.62 ± 0.12 mg dL^−1^, *p* = 0.753, n = 6; SYA@MIL-100(Fe)-10%: from 0.53 ± 0.02 to 0.64 ± 0.12 mg d L^−1^, *p* = 0.396, n = 6). These findings suggest that both pristine and formulated syringic acid exhibit hepatoprotective potential without adversely affecting renal function. The serological data indicate the absence of systemic toxicity at the biochemical level. Microscopic examination of hepatic tissues revealed signs of mild-to-moderate hepatocyte degeneration alongside bile duct proliferation. In parallel, renal samples displayed comparable levels of structural degeneration within both the glomeruli and tubules. Microscopic examination revealed signs of mild-to-moderate hepatocyte degeneration and bile duct proliferation. However, these structural observations did not correlate with the functional biochemical profile. In acute drug-induced liver injury, hepatocyte degeneration is typically accompanied by a sharp elevation in serum transaminases. In contrast, this study observed a significant reduction in ALT (*p* = 0.002) and AST (*p* = 0.016) levels. This functional improvement suggests that the observed histological changes are likely incidental background pathologies common in laboratory rodents, rather than indicative of acute, formulation-induced hepatotoxicity. The possible cause of the tissue damage might be the process or materials used during the preparation of the organs for mounting for histopathological analysis. Additionally, based on OECD 423 criteria, both the pristine drug and the MOF formulation are classified as having low acute oral toxicity.

### 3.10. Bioavailability

The pharmacokinetic profiles of free syringic acid and the SYA@MIL-100(Fe)-10% formulation were analyzed using a non-compartmental model. Table 3 summarizes the calculated parameters used to assess comparative bioavailability. No significant adverse effects were noted after the administration of the test compound. No data from the test animal were excluded in the data analysis and presentation. The mean plasma concentration–time profiles following oral and intraperitoneal (i.p.) administration are illustrated in Figure 10. For oral delivery, both preparations were administered at a dose equivalent to 100 mg kg^−1^ of syringic acid. In the i.p. groups, pristine syringic acid was administered at 25 mg kg^−1^, while the formulation was dosed at 1.99 mg kg^−1^ (syringic acid equivalent). To facilitate a direct comparison across different dosing levels, plasma concentration data were normalized to a standard 100 mg kg^−1^ equivalent dose. Systemic availability and circulatory retention were assessed by calculating the area under the curve (AUC). A detailed compilation of the resulting pharmacokinetic parameters is provided in Table 3.

**Figure 11:**
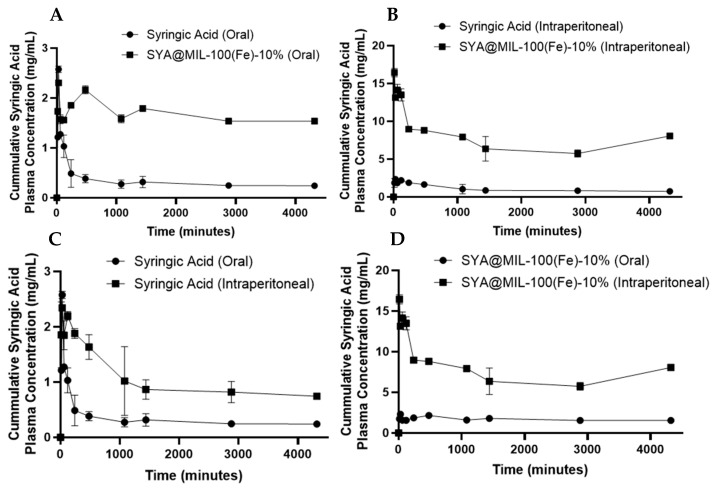
Plasma concentration–time profiles (AUC_0–720_) quantifying syringic acid levels in blood. The panels illustrate (**A**) oral administration (free drug vs. formulation); (**B**) intraperitoneal administration (free drug vs. formulation); (**C**) route comparison for pure syringic acid (oral vs. IP); and (**D**) route comparison for SYA@MIL-100(Fe)-10% (oral vs. IP). The *Y*-axis represents mean plasma concentration (mg/mL) plotted against time (min). Error bars denote S.E.M. (n = 3).

As shown in Figure 11A–D, the area under the curve for the 0 time to 72 h (AUC_0–72_) determined in the samples administered orally in the blood is significantly higher in SYA@MIL-100(Fe)-10% (7221.33 ± 97.63 mg min mL^−1^) compared to syringic acid (1419.00 ± 142.15 mg min mL^−1^) alone at a *p*-value of less than 0.001. Likewise, the AUC for the 0 time to infinity (AUC_0–∞_) showed a significant difference between SYA@MIL-100(Fe)-10% and syringic acid (7634.00 ± 97.60 mg min mL^−1^ and 1460.37 ± 143.84 mg min mL^−1^, respectively) at a *p*-value of 0.01. No significant differences were observed in maximum concentration (Cmax), the time to reach maximum concentration (T_max_), and elimination half-life (T_1/2_) between SYA@MIL-100(Fe)-10% (2.60 ± 0.01 mg mL^−1^, 52.02 ± 1.34 min, and 186.15 ± 2.62 min, respectively) and syringic acid (2.34 ± 0.19 mg mL^−1^ and 66.78 ± 7.56 min, and 118.77 ± 30.76 min, respectively), with *p*-values of 0.233, 0.127, and 0.094, respectively.

For the intraperitoneal route of administration using the blood samples, significant differences were noted with AUC_0–72,_ AUC_0–∞_, C_max_, and T_max_ at *p*-values of less than 0.001. SYA@MIL-100(Fe)-10% showed significantly higher pharmacokinetic parameters (31,297.33 ± 661.33 mg min mL^−1^, 42,846.43 ± 3703.76 mg min mL^−1^, 16.62 ± 0.44 mg mL^−1^, and 26.72 ± 1.03 min, respectively) compared to syringic acid alone (4368.33 ± 489.25 mg min mL^−1^, 5460.84 ± 964.81 mg min mL^−1^, 2.55 ± 0.04 mg mL^−1^, and 55.97 ± 2.11 min, respectively). No significant difference was noted on T_1/2_ for syringic acid (999.64 ± 410.34 min) and SYA@MIL-100(Fe)-10% (997.69 ± 377.83 min) at a *p*-value of 0.094.

**Figure 12:**
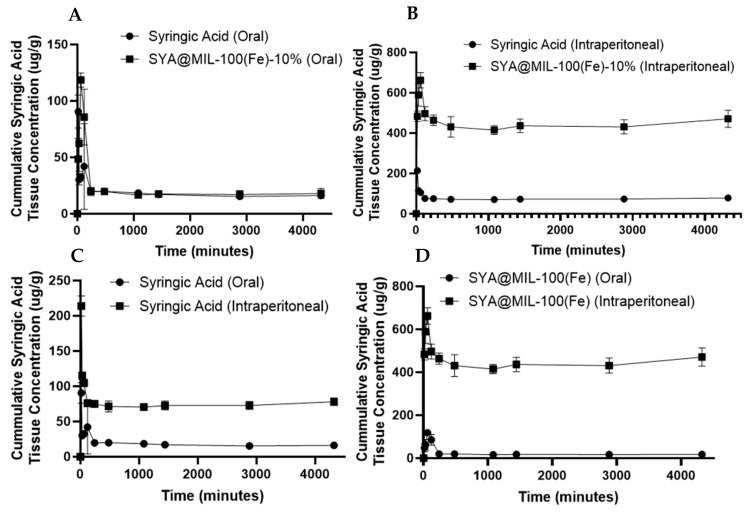
Kidney tissue concentration–time profiles (AUC_0–720_) quantifying syringic acid levels in tissue sample. The panels illustrate (**A**) oral administration (free drug vs. formulation); (**B**) intraperitoneal administration (free drug vs. formulation); (**C**) route comparison for pure syringic acid (oral vs. IP); and (**D**) route comparison for SYA@MIL-100(Fe)-10% (oral vs. IP). The *Y*-axis represents mean tissue concentration (µg/g) plotted against time (min). Error bars denote S.E.M. (n = 3).

The area under the curve for the 0 time to 72 h (AUC_0–72_) determined in the samples administered orally in the kidney showed no significant difference for SYA@MIL-100(Fe)-10% (88,202.33 ± 4,855.64 µg min g^−1^) compared to syringic acid (77,153.33 ± 2531.03 µg min g^−1^) at a p-value of 0.114 as shown in Figure 12A–D. In congruence, the AUC for the 0 time to infinity (AUC_0–∞_), the time to reach maximum concentration (T_max_), and the elimination half-life (T_1/2_) were significantly higher in SYA@MIL-100(Fe)-10% (93,818.14 ± 4364 µg min g^−1^, 94.54 ± 5.27 min, and 236.36 ± 71.08 min, respectively) compared to syringic acid alone (78,035.27 ± 2458 µg min g^−1^, 28.21 ± 0.33 min, 37.56 ± 4.69 min, respectively) at the *p*-values of 0.034, less than 0.001, and 0.049, respectively. No significant difference was noted on C_max_ for SYA@MIL-100(Fe)-10% (127.28 ± 5.96 µg g^−1^) and syringic acid (105.36 ± 9.79 µg g^−1^) at a *p*-value of 0.128.

For the intraperitoneal route of administration using the kidney samples, significant differences were noted with AUC_0–72_, C_max_, and T_max_ at *p*-values of less than 0.001; specifically, SYA@MIL 100 (Fe)-10% showed significantly higher pharmacokinetic parameters (1,911,750.00 ± 76,232.91 µg min g^−1^, 669.96 ± 26.14 µg g^−1^, and 94.96 ± 1.36 min, respectively) compared to syringic acid alone (321,104.67 ± 11,949.32 µg min g^−1^, 218.21 ± 8.73 µg g^−1^, and 24.15 ± 0.96 min, respectively). No significant differences were noted on AUC_0–∞_ and T_1/2_ for SYA@MIL-100(Fe)-10% (21,513,220.82 ± 16,644,903.64 µg min g^−1^, and 28,587.55 ± 24,474.46 min, respectively) and syringic acid (808,296.73 ± 429,472.17 µg min g^−1^, and 792.21 ± 153.25 min, respectively) at *p*-values of 0.282 and 0.320, respectively.

**Figure 13:**
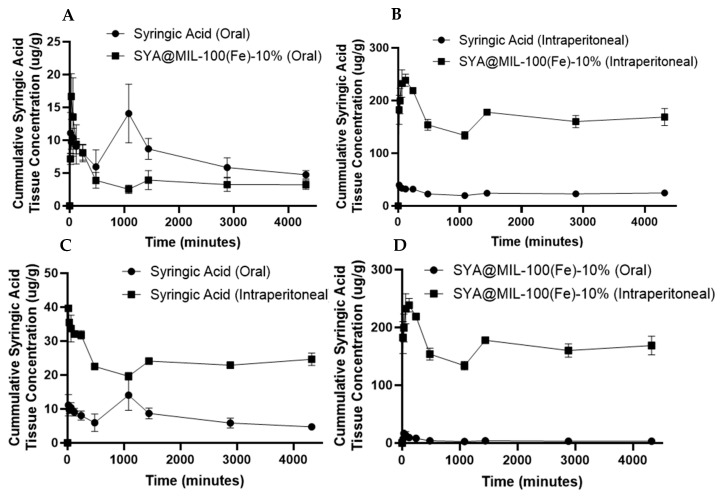
Liver tissue concentration–time profiles (AUC_0–720_) quantifying syringic acid levels in tissue sample. The panels illustrate (**A**) oral administration (free drug vs. formulation); (**B**) intraperitoneal administration (free drug vs. formulation); (**C**) route comparison for pure syringic acid (oral vs. IP); and (**D**) route comparison for SYA@MIL-100(Fe)-10% (oral vs. IP). The *Y*-axis represents mean tissue concentration (µg/g) plotted against time (min). Error bars denote S.E.M. (n = 3).

The area under the curve for the 0 time to 72 h (AUC_0–72_) determined in the samples administered orally in the liver is significantly higher in the syringic acid alone (32,000.33 ± 3544.16 µg min g^−1^) compared to SYA@MIL-100(Fe)-10% (16,754.33 ± 2268.79 µg min g^−1^) at a *p*-value of 0.022, as illustrated in Figure 13A–D. Likewise, the AUC for the 0 time to infinity (AUC_0−∞_) and the time to reach maximum concentration (T_max_) show significant differences between syringic acid (47,401.91 ± 8515.77 µg min g^−1^, and 515.98 ± 4.44 min, respectively) and SYA@MIL-100(Fe)-10% (17,264.74 ± 2353.25 µg min g^−1^, and 35.46 ± 4.39 min, respectively) at *p*-values of 0.027, less than 0.001, and 0.055. No significant difference was observed with the elimination half-life (T_1/2_) and the maximum concentration (C_max_) between SYA@MIL-100(Fe)-10% (110.08 ± 11.75 min, and 16.89 ± 1.84 µg g^−1^) and syringic acid (2288.81 ± 812.66 min, and 16.28 ± 3.54 µg g^−1^) at *p*-values of 0.055 and 0.89, respectively.

For the intraperitoneal route of administration using the liver samples, significant differences were noted with AUC_0–72_, C_max_, and T_max_ at *p*-values of less than 0.001. SYA@MIL 100 (Fe)-10% showed significantly higher pharmacokinetic parameters (719,560.00 ± 14,449.94 µg min g^−1^, 245.65 ± 10.86 µg g^−1^, and 110.54 ± 4.25 min, respectively) compared to syringic acid alone (102,784.67 ± 1510.75 µg min g^−1^, 40.13 ± 0.56 µg g^−1^, and 24.70 ± 0.21 min, respectively). No significant differences were noted on AUC_0–∞_ and T_1/2_ for SYA@MIL-100(Fe)-10% (2,893,510.76 ± 1,716,470.52 µg min g^−1^, and 8312.26 ± 6290.46 min, respectively) and syringic acid (166,522 ± 34,487.20 µg min g^−1^, and 1852.40 ± 1105.09 min, respectively) at *p*-values of 0.19 and 0.37, respectively.

## 4. Discussion

The assembly of metal–organic frameworks (MOFs) typically involves the self-assembly of metal ions (Lewis acids/electron acceptors) and organic linkers (Lewis bases/electron donors). Leveraging this principle, this study introduces the first synthesis of a syringic acid-doped MIL-100(Fe) via a mixed-linker protocol. Furthermore, it establishes the utility of this iron-based system as a specific carrier for syringic acid, a coherent application not previously explored in the literature. The parent MIL-100(Fe) framework employs trimesic acid as the organic linker, which provides three electron-donating carboxylic acid groups essential for establishing its three-dimensional crystalline network. The incorporation of drug molecules into MOFs for controlled delivery applications has been extensively explored in recent studies [14,19,26,27]. In this work, syringic acid was introduced as a co-linker at varying molar ratios relative to the total organic linker content. PXRD analysis revealed that syringic acid concentrations exceeding 10% molar resulted in framework collapse. The structural collapse observed at syringic acid concentrations exceeding 10% further substantiates its role as a competitive coordinating ligand. Unlike passive guest molecules, which typically saturate pore volume without destroying the host lattice, syringic acid actively competes with trimesic acid for coordination to the iron (III) centers. Because syringic acid is monotopic, its excessive incorporation compromises the 3D connectivity required to sustain the MIL-100 topology, leading to the observed loss of crystallinity.

This limitation arises primarily from the difference in linker topology: trimesic acid is tritopic, possessing three carboxylic acid groups that enable the formation of the 3D MIL-100(Fe) network, whereas syringic acid is monotopic, containing only one carboxylic acid group and therefore unable to sustain the same structural connectivity at higher substitution levels [28]. Although syringic acid also contains phenolic hydroxyl and methoxy groups, these substituents cannot provide the bridging coordination necessary to preserve the MIL-100(Fe) framework. Instead, they may participate in alternative coordination modes, such as phenolate binding, which promote the formation of amorphous solids or low-dimensional coordination polymers. Additionally, the phenolic hydroxyl group can competitively complex with the Fe^3+^ centers, further disrupting the framework integrity at elevated syringic acid concentrations [29]. Compared to the pristine framework, the optimized MIL-100(Fe)-10% variant displayed a distinct deep reddish-brown hue. This colorimetric shift is indicative of the formation of iron–phenolic coordination complexes, resulting from the chelation between Fe^3+^ metal nodes and the phenolic moieties of syringic acid. As illustrated in Figure 1, a dual-loading strategy was employed: syringic acid was first introduced as a linker during de novo synthesis (Figure 1A) and subsequently increased via post-synthesis impregnation (Figure 1B). Consequently, the total drug-loading capacity represents the sum of the structural linker and the physically adsorbed drug. Under optimal conditions (1:2 carrier-to-drug molar ratio; 36-hour incubation), the system achieved a maximal loading of 66.85% ± 0.004%, equivalent to approximately 0.67 mg of syringic acid per mg of carrier. The percent drug loading decreased as follows at 36.67% ± 0.01% (48 h) < 35.73% ± 0.01% (12 h) < 31.31% ± 0.01% (24 h). A lower percentage of drug loading was observed at a 1:1 molar ratio, namely, 33.70% ± 0.00% (36 h) > 24.05% ± 0.01% (24 h) > 21.52% ± 0.01% (12 h) > 15.20% ± 0.01% (48 h). With the given data of both 1:1 and 1:2 molar ratios, it can be concluded that syringic acid is impregnated in the carrier at 36 h. For the 1:1 molar ratio, the encapsulation efficiency displayed a distinct temporal dependency. While drug uptake improved steadily between 12 and 36 h, a significant reduction was recorded at the 48-hour mark. This sudden decline suggests a compromise in structural integrity, likely caused by the nucleophilic moieties of syringic acid destabilizing the framework during prolonged exposure [8,16,30]. These interactions likely promote ligand displacement, where syringic acid competes with the endogenous trimesic acid for coordination sites. Moreover, the coordination bonds formed between the iron centers and syringic acid may be thermodynamically inferior to those of the parent trimesate, due to the specific electronic effects of the phenolic hydroxyl and methoxy substituents. Consequently, replacing the original linker with a weaker bound molecule undermines the mechanical stability of the lattice, eventually leading to structural degradation. At the 1:2 molar ratio, the samples exhibited time-independent loading behavior. This can be ascribed to the incorporation of syringic acid in the mixed-linker system, which generated a more hydrophilic and defect-rich pore surface. The presence of these polar sites likely facilitated rapid adsorption of the drug within the first 12 h. However, with prolonged exposure, partial structural relaxation of the framework may have induced desorption or redistribution of the drug, resulting in lower apparent loading at 24 and 48 h [6,7]. Despite having some structural alterations, the 1:2 molar ratio of the carrier to syringic acid still resulted in a higher drug-loading capacity, which is in congruence with other reported research [8,16,31,32].

The superior encapsulation efficiency observed in MIL-100(Fe)-10% can be ascribed to a favorable shift in physicochemical characteristics and optimized host–guest dynamics relative to the unmodified material. As detailed in our prior research, the pristine MIL-100(Fe) scaffold is characterized by a high BET surface area (2028.35 m^2^ g^−1^), a total pore volume of 0.855916 cm^3^ g^−1^, and a pore width of 29.717 Å. This highly porous architecture provides an ideal template which, when functionalized via the mixed-linker strategy, significantly enhances the accommodation of syringic acid molecules.

It is crucial to distinguish this mixed-linker integration from a simple “ship-around-bottle” encapsulation effect. Had the syringic acid merely been physically entrapped during synthesis, the occupation of void space would be expected to reduce the effective pore width. Contrarily, the observed expansion of the pore diameter (from 29.7 Å to 49.6 Å) confirms that the drug molecules are not simply filling the voids but are structurally altering the framework architecture. This geometric expansion, coupled with the framework collapse observed at substitution ratios exceeding 10%, serves as definitive evidence that syringic acid acts as a defect-inducing pseudo-linker that creates missing-node apertures, rather than a passively trapped guest.

Modification of the framework via the mixed-linker approach resulted in significant shifts in textural characteristics. The BET surface area decreased to 1684.36 m^2^ g^−1^, and the total pore volume was reduced to 0.798689 cm^3^ g^−1^. Most notably, however, the pore width increased to 49.636 Å. This expansion in pore size is particularly significant for drug delivery, as it facilitates the entry and entrapment of syringic acid. Given that the molecular diameter of syringic acid is only 7.17 Å (determined via semi-empirical optimization and energy minimization using Chem3D Pro 12.0), the broadened pores provide ample space for efficient guest molecule diffusion and capture. Crucially, the observed expansion in pore width (from 29.717 Å to 49.636 Å) provides structural evidence of linker substitution rather than simple pore filling. In mixed-linker MOFs, the replacement of a tritopic linker (trimesic acid) with a monotopic one (syringic acid) creates missing coordination nodes, effectively generating “structural defects” or larger aperture voids. If syringic acid were merely physically adsorbed within the pores, a reduction in effective pore width would be expected due to steric hindrance; the observed expansion, conversely, supports its integration into the lattice framework.

In comparison with the previous data of MIL-100(Fe), a smaller pore width of 29.717 Å has provided good impregnation of syringic acid, thus implying that with a bigger pore width, the impregnation would result in an even more enhanced capacity [33,34]. Although the application of MIL-100(Fe) as a drug delivery system is well-documented, prior studies have predominantly focused on the encapsulation of high-molecular-weight compounds or bulky guest molecules, such as oxaliplatin [35], curcumin [36], cyclophosphamide [37], chloroquine [38], doxycycline, tetracycline [39], cephalexin [40], lamivudine [41], magnolol [16], and aceclofenac [42]. The expansion of pore width achieved via the mixed-linker strategy suggests that syringic acid—a relatively small molecule—faces reduced steric hindrance during encapsulation. This structural modification rationalizes the superior drug-loading capacity of MIL-100(Fe)-10% relative to the pristine framework. Furthermore, the internal microenvironment of the MOF is inherently amphiphilic. This dual characteristic is crucial, as it creates a versatile host environment capable of accommodating and stabilizing both hydrophilic and hydrophobic guest molecules [8,16,43].

The superior drug-loading capacity (66.85%) observed in this study is attributed to a synergistic dual-incorporation strategy. Unlike conventional loading, which relies solely on physical adsorption, this approach utilizes syringic acid in two distinct roles, as a structural defect inducer and as a physical guest. As a structural defect inducer, during the initial de novo synthesis, syringic acid acts as a competitive co-linker. Its substitution for trimesic acid creates “missing-node” defects, which significantly expands the pore width from 29.717 Å to 49.636 Å. As a physical guest, in the subsequent impregnation phase, this expanded pore architecture reduces steric hindrance, allowing a significantly higher volume of additional syringic acid to be physically adsorbed within the mesoporous cages.

Consequently, the final formulation comprises both chemically coordinated drug molecules (embedded in the framework lattice) and physically adsorbed guest molecules (occupying the void spaces), resulting in a maximal loading capacity that significantly outperforms the pristine framework.

**Figure 14:**
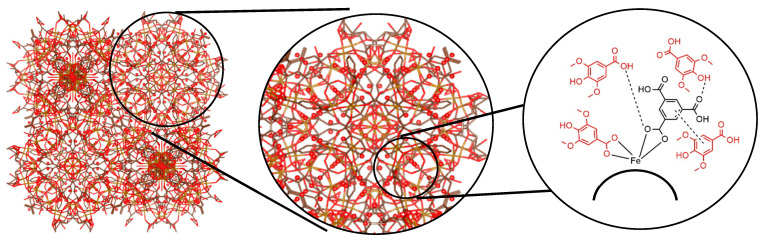
Proposed binding mechanism of syringic acid within the MIL-100(Fe)-10% lattice, highlighting stabilization via hydrogen bonding, π-π stacking, and metal–ligand coordination.

The sequestration of syringic acid within the MIL-100(Fe)-10% matrix is governed by a multifaceted interplay of non-covalent forces and coordination chemistry (Figure 14). Primary stabilization mechanisms likely include hydrogen bonding, π-π stacking, and direct metal–ligand complexation. Specifically, hydrogen bonds are postulated to form between the hydroxyl/carboxyl groups of the guest molecule and the carboxylic moieties of the trimesic acid linkers. Notably, the mixed-linker architecture introduces structural syringic acid, providing supplementary free hydroxyl sites that further enhance H-bonding potential. Simultaneously, π-π stacking interactions are expected between the aromatic ring of the guest drug and the conjugated systems of the organic framework. Evidence of direct coordination between the Fe centers and the nucleophilic oxygen of syringic acid is provided by the macroscopic color deepening—from bluish-brown to a darker shade—observed post-loading. Finally, electrostatic forces within the amphiphilic pore environment contribute to the overall retention of the guest [44].

Although previous investigations utilizing mPEG-PLGA-PLL nanoparticles [45], SMEDDS [23], and TPGS liposomes [2] reported superior entrapment efficiencies (EEs) ranging from 92.69% to 98.04%, these studies notably failed to disclose percent drug loading (DL)—a vital parameter for determining the actual payload capacity of a nanocarrier. In contrast, this work establishes a clear advantage in loading capacity. The modified MIL-100(Fe)-10% achieved a DL of 66.85 ± 0.004%, which was significantly higher than the 64.42 ± 0.03% observed for the pristine framework (*p* < 0.001). Statistical evaluation via two-way ANOVA confirmed that both the incubation period (12 vs. 36 h) and the type of framework (mixed-linker vs. pristine) are significant determinants of drug capacity (*p* < 0.001). These results corroborate studies in the existing literature stating that extending contact time improves impregnation up to a saturation threshold, after which framework stability may be compromised [8,16]. Additionally, the superior performance of the 1:2 ratio over the 1:1 formulation supports the principle that a higher initial concentration gradient drives more efficient encapsulation [46].

Analysis of the PXRD patterns revealed notable fluctuations in Bragg peak intensities following the integration of syringic acid via the mixed-linker strategy and subsequent impregnation. These intensity variations serve as a diagnostic indicator of host–guest interactions within the MIL-100(Fe)-10% matrix. The redistribution of diffraction intensities confirms successful drug encapsulation, as the presence of guest molecules perturbs the electron density distribution within the unit cell. This perturbation fundamentally alters the structure factors (F_hkl_) corresponding to specific crystallographic planes. Crucially, while the peak intensities shifted, the Bragg angles remained constant, verifying that the crystalline order and framework integrity of the carrier were preserved.

These findings are consistent with the previously reported studies on MIL-100(Fe) systems, wherein the encapsulation of guest molecules—such as 5-fluorouracil or caffeine—likewise produced a redistribution of key peak intensities (e.g., variations in the (022):(357) ratio) without any observable shift in diffraction angles. Such behavior has been attributed to electron density rearrangements caused by guest–framework interactions, resulting in modified structure factors but retaining full crystallinity of the host lattice [47].

The deviation between the theoretical and experimental PXRD profiles—specifically regarding the relative intensities of the (220) and (311) reflections—is consistent with well-established observations in porous framework materials. Such discrepancies are typically attributed to the occupancy of pore channels by residual guest species. While the simulated pattern assumes an idealized, completely evacuated structure, the as-synthesized MIL-100(Fe) and MIL-100(Fe)-10% samples often retain trace amounts of solvent or moisture even after activation. Analogous to the effect of syringic acid loading, these residual molecules perturb the electron density within the cavities. This alteration affects the scattering contrast of the pores, resulting in the observed intensity variations relative to the guest-free model [43].

Given the substantial structural resemblance between the functional moieties of syringic acid and the trimesic acid linkers, FTIR analysis exhibited only negligible spectral deviations post-loading. The vibrational signatures of the drug were largely obscured by the dominant signals of the framework, particularly in the aromatic and carboxylate regions. This overlap is consistent with the mixed-linker design; since syringic acid is already integral to the MIL-100(Fe)-10% lattice, subsequent impregnation induces minimal additional spectral changes. Moreover, FTIR lacks the spatial resolution to differentiate between internal pore sequestration and exterior surface adsorption. Consequently, while informative, spectroscopy alone is insufficient. To definitively confirm intrapore encapsulation and rule out surface phenomena, orthogonal characterization techniques—specifically BET porosity measurements and PXRD—were utilized.

Post-loading analysis indicated a universal decline in total pore volume, confirming that syringic acid successfully occupies the framework’s void space. Interestingly, the micropore volume exhibited a consistent upward trend across all time points. This finding diverges from the standard literature on drug-loaded MIL-100(Fe), where guest inclusion typically triggers micropore blockage. The observed expansion implies that the drug is not spatially restricted to the micropores.

Rather than signaling drug leakage, this phenomenon is likely attributable to dynamic structural changes, such as the elution of residual solvent, “breathing” of the flexible lattice, or minor structural rearrangements. Furthermore, mechanisms like partial etching or internal diffusion may have re-opened access to the microporous domains. Therefore, this recovery of micropore volume signifies improved internal accessibility rather than reduced loading efficiency.

Crucially, the simultaneous decrease in mesoporous volume provides robust evidence that syringic acid is preferentially sequestered within the larger mesoporous cages of the MIL-100(Fe) hierarchy. The most significant volume reduction occurred at 36 h, correlating directly with the maximal drug-loading capacity recorded. This suggests that the drug molecules predominantly occupy the large internal cages—which offer sufficient steric space for bulkier guests—after traversing the smaller microporous windows. As molecules diffuse deeper into these mesoporous sanctuaries over time, the micropore entrances are cleared, explaining the partial recovery of micropore volume. While rapid impregnation was observed between 12 and 36 h, the slight reversal at 48 h may be attributed to desorption, guest reorganization, or framework relaxation. Overall, these findings confirm that the 7.17 Å syringic acid molecule fits within the pore architecture, supporting a mechanism of true internal encapsulation rather than surface adsorption.

Post-impregnation analysis revealed a notable expansion in pore width. This phenomenon is likely attributable to structural relaxation or partial swelling of the lattice, driven by specific host–guest interactions between the phenolic moieties of syringic acid and the coordinatively unsaturated iron centers of the MIL-100(Fe)-10% nodes. Alternatively, this increase may result from the displacement of residual solvent or adsorbed species during the loading phase; removing these obstructions would effectively reopen constricted channels, thereby increasing the average pore width calculated via the BJH method. These observations are consistent with the inherent structural flexibility (“breathing” effect) characteristic of the MIL-100(Fe) family. While this indicates successful guest accommodation and minor geometric adjustments, it is important to acknowledge that nitrogen adsorption data can be sensitive to framework dynamics and residual solvent content, potentially influencing the precision of these textural parameters.

Thermogravimetric analysis revealed that the thermal stability of the mixed-linker MIL-100(Fe)-10% is virtually indistinguishable from that of the pristine MIL-100(Fe), with both materials undergoing decomposition in the 300–350 °C range. This indicates that integrating syringic acid into the framework lattice did not compromise its thermal resistance. However, a significant deviation in the degradation profile was noted following the physical impregnation of the drug. In the drug-loaded samples, the onset of primary decomposition shifted to approximately 500 °C, followed by a secondary mass loss event near 700 °C. This substantial increase in thermal stability implies the formation of robust host–guest interactions between the encapsulated syringic acid and the metal–organic scaffold. Physically confined syringic acid likely interacts with open Fe sites and pore surfaces through hydrogen bonding or π–π interactions, which enhance the thermal resistance of the structure. Moreover, during heating, syringic acid may undergo partial carbonization, forming a protective char layer that further delays oxidative degradation. The absence of a distinct melting endotherm for syringic acid (205–209 °C) in the TGA curve supports this interpretation, implying strong confinement and restricted molecular mobility within the pores. The findings demonstrate that impregnation alters the decomposition kinetics and bolsters framework stability through combined coordination and passivation effects [48]. This represents a novel divergence from the prior literature; specifically, the enhanced stability observed here is exclusive to the SYA@MIL-100(Fe)-10% formulation and was not detected in the earlier SYA@MIL-100(Fe) study [8].

The residual weights in MIL-100(Fe) (30.08%) compared to the MIL-100(Fe)-10% (28.84%) may be due to the greater amount of organic compound present in the molecule, as syringic acid was used as an organic linker through a mixed-linker approach. Minor fluctuations were noted with SYA@MIL-100(Fe)-10% at 12 h (27.51%) and 24 h (29.77%), as the pores and void spaces were not yet filled up with syringic acid. At the highest drug loading, a residual weight of 35.67% was noted, probably due to a stronger host–guest interaction (Fe-O phenolic interaction), which can result in a higher yield of the residual compound, Fe_2_O_3_. At 48 h, the residual weight was recorded at 24.43%, the lowest residual weight among all the test samples, which is probably due to framework degradation or collapse.

The increased residual mass observed in SYA@MIL-100(Fe)-10% relative to the unloaded MIL-100(Fe)-10% stems from complex interactions between the syringic acid guest and the metal–organic host. Firstly, syringic acid, being an aromatic phenolic compound, tends to undergo carbonization during thermal decomposition, forming a stable carbonaceous char that increases the residual mass. Secondly, the encapsulation of syringic acid within the porous network likely impedes the oxidative breakdown of the organic linkers. By functioning as a protective shield or coating, the guest molecules effectively retard the kinetics of the overall combustion process. Additionally, strong coordination interactions between syringic acid and the open Fe sites could lead to the formation of thermally stable organometallic complexes, contributing to a higher inorganic residue (e.g., Fe_2_O_3_) upon heating. Another possibility is that pore-blocking effects from loaded syringic acid restrict the diffusion of volatile decomposition products, leading to incomplete mass loss within the TGA temperature range. Collectively, these factors—carbon char formation, oxidative shielding, metal coordination, and diffusion limitation—account for the higher residual mass observed in SYA@MIL-100(Fe)-10%.

Thermal analysis revealed a distinct shift in the decomposition profile of the drug-loaded framework compared to its empty counterpart. While MIL-100(Fe)-10% exhibited a degradation onset at approximately 300 °C, the SYA@MIL-100(Fe) formulation degraded between 253 and 263 °C. The characteristic melting endotherm of pristine syringic acid (205–209 °C) shifted to a higher temperature range in the composite material. This observation implies that the confinement within the framework effectively enhances the thermal stability of the drug through host–guest interactions. This phenomenon aligns with findings by Mileo et al. [49], regarding the confinement effects of porous matrices on guest molecules. Furthermore, the residual weight analysis provided quantitative evidence of drug loading. The empty MIL-100(Fe) retained approximately 30% of its original mass, corresponding to the formation of inorganic iron (III) oxide (Fe_2_O_3_). In contrast, the SYA@MIL-100(Fe) samples exhibited a lower residual weight (<30%). This decrease indicates a higher initial ratio of combustible organic material, confirming the substantial presence of syringic acid within the framework [50].

Enhancing solubility, bioavailability, and blood–brain barrier penetration are key advantages of nanoparticle-based delivery systems [51,52], with efficiency often correlated with smaller particle sizes and increased surface area [53]. In this study, MIL-100(Fe)-10% was presented as octahedral nanoparticles ranging from 100 to 300 nm. This physical profile mirrors that of the parent MIL-100(Fe) and supports cellular entry through specific endocytic mechanisms, namely clathrin- and caveolin-mediated pathways [54]. The octahedral shape is functionally critical; prior research indicates that this morphology promotes efficient uptake, specifically triggering clathrin-mediated endocytosis [55]. These structural properties validate the continued application of MIL-100(Fe)-10% as a robust drug carrier [5].

Utilization of the mixed-linker strategy significantly augmented the release profile of syringic acid compared to the unmodified scaffold. After 30 h, MIL-100(Fe)-10% released 54.97% ± 0.37% in water, 61.52% ± 0.93% in PBS (pH 7.4), 64.65% ± 0.55% in PBS (pH 6.8), and 68.23% ± 0.42% in 0.1 N HCl. These values represent a marked improvement over the pristine MIL-100(Fe) reported by Santos et al. (2025) [8], which achieved only 5.57%, 27.57%, 55.42%, and 25.80% in the respective media over the same period.

The rapid elution to the destabilizing influence of nucleophilic species within the media was observed [8,16,56,57,58]. Specifically, phosphate ions (in PBS) compete with trimesic acid for iron coordination, effectively displacing the linker, while chloride ions (in HCl) induce protonation of the carboxylate groups, triggering irreversible structural collapse [16]. Since these ions are abundant in the bloodstream, this suggests a viable mechanism for systemic release. Furthermore, the incorporation of syringic acid likely reduces the framework dimensionality (potentially from 3D to 2D or 1D) [28] and introduces steric hindrance. This steric bulk weakens interfacial connectivity and coordination bonds [59], ultimately reducing overall stability. Despite these structural alterations, the carrier maintained a pH-sensitive release pattern comparable to the parent material, favoring release in PBS pH 7.4 over pH 6.8.

To determine the kinetic mechanism underlying syringic acid elution, the experimental data were analyzed using five distinct mathematical models. For aqueous and acidic environments (0.1N HCl), the Korsmeyer–Peppas equation yielded the highest correlation coefficients (r^2^ = 0.9861 and 0.9998, respectively). Conversely, release behavior in phosphate-buffered systems varied: the profile in PBS pH 7.4 was best approximated by the Higuchi model (r^2^ = 0.8579), whereas the data in PBS pH 6.8 adhered strictly to first-order kinetics (r^2^ = 0.9998). Evaluation of the diffusional exponent (n) derived from the Korsmeyer–Peppas model showed values well below the Fickian threshold of 0.45 for both water (n = 0.0373) and simulated gastric fluid (n = 0.0114). These results are indicative of Quasi-Fickian diffusion, implying that drug transport is driven by passive diffusion through the rigid porous network rather than by matrix swelling or relaxation. The exceptionally low n-values further suggest that diffusion is physically hindered, likely due to tight host–guest confinement within the micropores [8,16].

This behavior mirrors findings in other rigid porous systems, such as Zn-based MOFs, where restricted diffusion leads to similarly low exponents [60,61,62]. Additionally, the conformity of the pH 7.4 data to the Higuchi model reinforces the conclusion that the mechanism is diffusion-controlled. This model is predicated on Fickian release from a non-swelling matrix, which is characteristic of stable metal–organic frameworks. This interpretation is supported by analogous reports on MOF-mediated delivery, including ketoprofen from Sr/PTA MOF [63], ibuprofen from UiO-66-NH_2_ [64], and doxorubicin from Cu_3_(BTC)_2_ [65]. Similar diffusion-driven dynamics have also been documented for nanocomposites [66] and bio-hybrids [67], collectively validating the capacity of MIL-100(Fe)-10% to function as a stable, diffusion-regulated delivery vehicle.

The elution profile of SYA@MIL-100(Fe) was best approximated by the first-order kinetic model, indicating that the release rate is directly proportional to the concentration of the remaining drug. As the payload within the matrix depletes, the rate of release diminishes over time. This kinetic signature is characteristic of systems governed primarily by diffusion, where the matrix successfully prevents a rapid initial burst. The MIL-100(Fe) scaffold thus functions as a controlled reservoir, facilitating the gradual diffusion of syringic acid into the surrounding medium.

This concentration-dependent behavior aligns with numerous studies on MOF-based delivery. For instance, similar first-order release patterns have been documented for folic acid from CD-MOF@SiO_2_ nanocomposite [66], ibuprofen and captopril from Zr-MOF [68], magnesium ion from GR-MOF-27 [69], and 5-fluorouracil and curcumin from MIL-88B [70]. These precedents reinforce the utility of the first-order model in describing prolonged drug transport from porous coordination polymers.

In accordance with OECD 423 protocols, the acute oral toxicity assessment at 2000 mg kg^−1^ demonstrated that both the pure drug and the loaded carrier maintain a safety profile comparable to their individual constituents. Syringic acid is generally not classified as an acute toxin according to Safe Work Australia, with prior studies reporting no toxic effects even at daily doses of 1000 mg kg^−1^ over 14 days [71]. Likewise, the MIL-100(Fe) scaffold exhibits a favorable biological safety profile. The literature confirms its cytocompatibility, reporting negligible toxicity towards both normal human hepatic cells (HL-7702) and hepatocellular carcinoma models (HepG2) [72] [73].

From a mechanistic perspective, the biodegradation of the metal–organic framework is likely facilitated by the physiological environment. Previous research indicates that the presence of strongly nucleophilic biomolecules, such as proteins and phosphate groups, can trigger framework collapse. These nucleophiles effectively compete with the organic linkers for coordination to the iron centers, destabilizing the lattice. This process results in the release of water-soluble secondary building unit (SBU) complexes, which are readily cleared from the system [8,16].

Consistent with the findings obtained using the parent MIL-100(Fe) system, the serological data from the acute oral toxicity study of SYA@MIL-100(Fe)-10% corroborate the well-documented hepatoprotective properties of syringic acid, which include antioxidant activity, inhibition of lipid peroxidation, and modulation of hepatic inflammatory responses. Notably, the more pronounced reduction in liver enzyme levels observed with the MIL-100(Fe)-10% formulation suggests an additional therapeutic advantage arising from the sustained and targeted delivery facilitated by the MOF matrix. As a modified derivative of MIL-100(Fe), the MIL-100(Fe)-10% framework likely improved the tissue distribution and retention of syringic acid at hepatocellular sites. This is supported by our previously published findings on SYA@MIL-100(Fe), where quantifiable levels of syringic acid were detected in liver tissues of rat models during bioavailability studies.

Compared to the free form of syringic acid, which is limited by poor aqueous solubility and rapid systemic elimination, the SYA@MIL-100(Fe)-10% formulation demonstrated slower systemic clearance and enhanced bioavailability. This was evidenced by the pharmacokinetic data, particularly the significantly higher AUC values for syringic acid in liver tissue relative to plasma (Table 3). Such prolonged hepatic exposure likely promoted sustained antioxidant activity, minimizing oxidative stress and hepatocellular injury. The observed reductions in AST and ALT levels therefore not only reaffirm the intrinsic hepatoprotective effects of syringic acid, as reported in CCl_4_-, APAP-, thioacetamide-, and NAFLD-induced models [74,75,76,77], but also underscore the effectiveness of MIL-100(Fe) as a nanocarrier for enhancing the therapeutic potential of natural phenolic compounds.

Importantly, the integration of syringic acid into the MIL-100(Fe)-10% framework did not compromise its biocompatibility. Only minimal hepatic changes, such as mild hepatocellular degeneration, were observed, with no signs of severe toxicity or irreversible damage. These results suggest that SYA@MIL-100(Fe)-10% remains safe for oral administration within the tested dose of 2000 mg·kg^−1^, aligning with established safety profiles of syringic acid.

The serological data obtained from the acute oral toxicity assessment of SYA@MIL-100(Fe)-10% align with the findings for the parent MIL-100(Fe) system, corroborating the established hepatoprotective profile of syringic acid, namely its antioxidant capacity, inhibition of lipid peroxidation, and modulation of hepatic inflammation. Notably, the formulation elicited a more profound reduction in liver enzyme levels compared to the free drug, suggesting a synergistic therapeutic advantage driven by the sustained delivery capabilities of the MOF matrix. As a derivative of the MIL-100(Fe) family, the modified framework appears to enhance the tissue distribution and retention of the payload at hepatocellular sites. This hypothesis is substantiated by our previous bioavailability studies, which detected quantifiable concentrations of syringic acid in the liver tissues of rat models following administration of SYA@MIL-100(Fe).

Relative to free syringic acid, which is constrained by poor aqueous solubility and rapid elimination, the SYA@MIL-100(Fe)-10% formulation exhibited slower systemic clearance and superior bioavailability. Pharmacokinetic analysis revealed significantly higher AUC values in liver tissue compared to plasma (Table 3), indicating prolonged hepatic exposure. This extended residence time likely facilitates sustained antioxidant activity, thereby effectively mitigating oxidative stress and hepatocellular injury. Consequently, the observed reductions in AST and ALT levels not only validate the intrinsic hepatoprotective efficacy of syringic acid—as reported in various induced-injury models (e.g., CCl_4_, APAP, thioacetamide, NAFLD) [74,75,76,77]—but also highlight the utility of MIL-100(Fe) as a nanocarrier for optimizing the therapeutic index of phenolic compounds. Crucially, the integration of the drug did not compromise biocompatibility; only mild, reversible hepatic changes were noted, confirming that SYA@MIL-100(Fe)-10% remains safe for oral administration at the 2000 mg kg^−1^ limit dose.

Furthermore, syringic acid’s nephroprotective properties were evident in the lowered levels of key renal biomarkers, including BUN and creatinine, consistent with previous findings in models of diabetic nephropathy [78,79], chronic hyperglycemia-induced renal injury [80], and oxidative mitochondrial stress [81]. These findings collectively indicate that the MIL-100(Fe)-10% formulation supports both hepatic and renal safety, reaffirming its potential as a safe and effective vehicle for the sustained delivery of phenolic antioxidants.

Encapsulation within the MIL-100(Fe)-10% matrix resulted in a substantial improvement in the pharmacokinetic behavior of syringic acid. Analysis of the area under the curve AUC_0–72_ revealed that the nano-formulation significantly outperformed the free drug across both administration routes. Specifically, oral administration yielded an AUC of 7221.33 ± 97.63 mg min mL^−1^ compared to 1419 ± 142.15 mg min mL^−1^ for the free compound. Similarly, intraperitoneal delivery showed a marked increase to 31,297.33 ± 661.33 mg min mL^−1^ versus 4368.33 ± 489.25 mg min mL-^1^. These enhancements correspond to relative bioavailability F_rel_ values of 5.09 and 7.17 for the oral and intraperitoneal pathways, respectively

When calculated using AUC_0–∞_, the relative bioavailability (F_rel_) increased to 5.22 for the oral route and 7.85 for the intraperitoneal route, further attributing the enhanced absorption to the MIL-100(Fe)-10% carrier. Although these values are lower than those obtained with unmodified MIL-100(Fe), the formulation successfully meets the study’s primary objective of improving oral bioavailability. Notably, the oral SYA@MIL-100(Fe)-10% demonstrated an F_rel_ of 1.65 compared to intraperitoneally administered free syringic acid AUC_0–72_. This suggests that the MIL-100(Fe)-10% carrier can effectively replace parenteral administration when the goal is to maximize systemic drug availability.

A similar trend was observed using AUC_0–∞_, which yielded a ratio of 1.40 for the intraperitoneal SYA@MIL-100(Fe)-10% compared to free syringic acid, a finding consistent with the previous literature [16,82]. Non-compartmental analysis revealed that the formulation significantly extended the elimination half-life via the oral route (186.15 ± 2.62 min) compared to oral free syringic acid (118.77 ± 30.76 min). Conversely, no significant difference was observed via the intraperitoneal route (997.69 ± 377.83 min vs. 999.64 ± 410 min). This prolonged retention serves as confirmation of the system’s slow, sustained-release mechanism. Consequently, the formulation may allow for reduced administration frequency while upholding therapeutic efficacy. By lowering the dosing burden and mitigating potential side effects, the system addresses critical barriers to success in chronic therapy—namely, patient safety and compliance.

Consequently, the extended elimination half-life serves as confirmation of the sustained-release characteristics intrinsic to SYA@MIL-100(Fe)-10%. Clinically, this translates to tangible benefits, including an improved safety profile, enhanced patient convenience through reduced dosing frequency, and the maintenance of consistent therapeutic plasma levels [47,83,84]. Notably, however, the elimination half-life of the modified SYA@MIL-100(Fe)-10% formulation was found to be markedly reduced compared to the unmodified SYA@MIL-100(Fe) system. This reduction aligns with in vitro data demonstrating a higher release rate for the 10% formulation. It suggests that the mixed-linker approach yields a material with lower dynamic stability, which is more prone to structural collapse, thereby facilitating a faster release of syringic acid compared to the pure framework.

Consistent with the elimination half-life findings, the T_max_ for SYA@MIL-100(Fe)-10% was significantly shorter than that of free syringic acid in both the oral (52.02 ± 1.34 min vs. 66.78 ± 7.56 min) and intraperitoneal (66.78 ± 7.56 min vs. 55.97 ± 2.11 min) routes. This reduced T_max_ reflects the impact of the mixed-linker approach, which introduces structural defects into the MIL-100(Fe) framework. While the modified material retains certain original properties, its pharmacokinetic behavior is significantly altered. Notably, the T_max_ for the intraperitoneal formulation was shorter than that of the oral administration. The rapid attainment of peak plasma concentration is likely driven by the high density of nucleophilic species within the peritoneal cavity and systemic circulation. Unlike the gastrointestinal environment, these biological compartments facilitate accelerated lattice disintegration, thereby expediting the release of the cargo.

The SYA@MIL-100(Fe)-10% formulation demonstrated a significant increase in C_max_ across both routes compared to syringic acid alone (oral: 16.62 ± 0.44 mg mL^−1^ vs. 2.33 ± 0.19 mg mL^−1^; IP: 16.89 ± 1.84 mg mL^−1^ vs. 2.55 ± 0.04 mg mL^−1^). When combined with the T_max_ results, a distinct pharmacokinetic profile emerges. Unlike the unmodified MIL-100(Fe), which is characterized by a longer T_max_ and sustained release, the mixed-linker approach yielded a derivative prone to a “burst release” effect in the early stages of administration. Bioavailability was augmented despite the accelerated release profile. This is attributed to the framework’s ability to preserve the drug’s integrity within the GI tract and facilitate its passage across biological barriers. Specifically, the nanoparticles likely enhance absorption by activating the clathrin-dependent endocytic pathway. Similar improvements in systemic availability were reported by Santos et al. using UiO-66(Zr) as a carrier for magnolol [12]. Furthermore, the relative bioavailability (F_rel_) achieved with SYA@MIL-100(Fe)-10% surpasses that of other syringic acid delivery systems, including TPGS/F127/F68 (2.3-fold) [85]; TPGS (2.8-fold) [2]; and SMEDDS (2.1-fold) [23].

Generally, the SYA@MIL-100(Fe)-10% formulation yielded significantly higher AUC_0–72_ and AUC_0–∞_ values in liver and kidney tissues compared to syringic acid alone, across both administration routes. A notable exception was observed in the liver following oral administration, where the free syringic acid exhibited a higher AUC_0–72_ 47,401.91 ± 8515.77 mg min µg^−1^ compared to the MOF formulation (16,754.33 ± 2268.79 mg min µg^−1^). This distribution pattern was corroborated by the AUC_0–∞_ values. In general, renal tissues exhibited significantly higher exposure levels compared to hepatic samples. This disparity implies a preferential accumulation in the kidneys, potentially driven by specific active transport systems or enhanced tissue retention within the renal parenchyma. The observed accumulation pattern mirrors prior reports on phenolic biodistribution. These compounds exhibit a predilection for high-perfusion, metabolically active sites, such as the renal and hepatic systems, reflecting the critical function of these organs in metabolic processing and excretion [86,87,88]. Furthermore, the elevated tissue concentrations with the MIL-100(Fe)-10% carrier may be attributed to internalization by hepatic and renal phagocytes. Once intracellular, the MOF structure degrades in the presence of nucleophilic moieties, such as phosphates, thereby releasing the drug [89].

The study utilized a minimal sample size (n = 3) per time point to strictly adhere to the 3Rs principle (Reduction) and institutional ethical mandates. It is acknowledged that this limited sample size contributed to the higher standard deviations observed in the pharmacokinetic parameters and constrained the precision of the population estimates. However, despite this variability, the improvement in bioavailability elicited by the SYA@MIL-100(Fe)-10% formulation was of sufficient magnitude to achieve statistical significance (*p* < 0.05) across primary metrics (AUC and Cmax) compared to the free drug. This indicates that the therapeutic effect size is substantial enough to be discerned even within a minimal cohort. Furthermore, while the Sprague Dawley rat is a standard model, species-specific physiological differences—such as the absence of a gallbladder and distinct hepatic enzymatic pathways—may alter the solubility and metabolic profile of syringic acid compared to humans, thereby limiting direct translational applicability. Additionally, although randomization and environmental standardization were employed to minimize confounders, the distinct physical appearance of the nanocarrier formulation compared to the free drug solution precluded complete blinding during administration, potentially introducing performance bias. Finally, the proposed mechanism of clathrin-mediated endocytosis remains an inference based on pharmacokinetic behavior and the literature rather than being definitively confirmed through specific cellular inhibition studies within this experimental cohort.

## 5. Conclusions

This study successfully synthesized a novel mixed-linker metal–organic framework, MIL-100(Fe)-10%, by substituting trimesic acid with syringic acid. The results confirm that while the substitution is stoichiometrically limited to 10% to preserve crystallinity, it induces a “defect engineering” effect that significantly expands pore width (from ~29 Å to ~49 Å), thereby reducing steric hindrance for guest molecules. This structural modification facilitated a superior drug-loading capacity of 66.85%, significantly outperforming the unmodified framework. In vivo evaluations demonstrated that the SYA@MIL-100(Fe)-10% formulation is not only biocompatible but also hepatoprotective, evidenced by statistically significant reductions in ALT and AST levels compared to baseline. Pharmacokinetically, the formulation successfully overcame the poor solubility of the free drug, achieving a 5.09-fold increase in relative oral bioavailability. Although the mixed-linker modification resulted in a faster release rate compared to the pristine scaffold due to altered host–guest interactions, it established a robust, diffusion-controlled delivery system (Quasi-Fickian kinetics). These findings validate the mixed-linker MIL-100(Fe)-10% as a potent, safe, and effective nanocarrier for enhancing the systemic performance of poorly soluble phenolic acids.

## 6. Patents

An ongoing patent application is being applied for in relation to this study.

## Figures and Tables

**Table 1:** Summary of the amount of the organic linker used in the preparation.

Label	Percent of Syringic Acid	Organic Linker *			
		Syringic Acid		Trimesic Acid	
		mmol	mg	mmol	mg
MIL-100(Fe)-10%	10%	0.079	15.655	0.711	149.410
MIL-100(Fe)-20%	20%	0.158	31.311	0.632	132.808
MIL-100(Fe)-30%	30%	0.237	46.966	0.553	116.207
MIL-100(Fe)-40%	40%	0.316	62.622	0.474	99.606
MIL-100(Fe)-50%	50%	0.395	78.277	0.395	83.005
MIL-100(Fe)-100%	100%	0.79	156.554	0	0.000

* The total amount of organic linker is based on the works of Luo and co-workers [6] as 0.79 mmol; molecular weight of syringic acid—198.17 g/mol; trimesic acid—210.14 g/mol.

**Table 2:** Summary of the textural characteristics of MIL-100(Fe), MIL-100(Fe)-10%, and SYA@MIL-100(Fe)-10% XXhrs.

Sample	BET Surface (m^2^ g^−1^)	Total Pore Volume (cm^3^ g^−1^) *	Micropore Volume (cm^3^ g^−1^) **	Mesopore Volume (cm^3^ g^−1^) ***	Pore Width (Å) ****
MIL-100(Fe)	2028.35	0.855916	0.297397	0.558519	29.717
MIL-100(Fe)-10%	1684.36	0.798689	0.267967	0.530722	49.636
SYA@MIL-100(Fe)-10%—12 h	1237.67	0.591158	0.340373	0.250785	60.229
SYA@MIL-100(Fe)-10%—24 h	1294.45	0.62355	0.366032	0.257518	59.689
SYA@MIL-100(Fe)-10%—36 h	1194.49	0.56121	0.336622	0.224588	58.936
SYA@MIL-100(Fe)-10%—48 h	1466.20	0.707342	0.407326	0.300016	58.178

* Single-point adsorption total pore volume of pores less than 3873.040 Å width at P/P_0_ = 0.995000000; **—t-plot 422 micropore volume; ***—calculated by subtracting the total pore volume from the micropore volume; ****—BJH 423 adsorption average pore width (4V/A).

**Table 3:** Comparative pharmacokinetic profile of free syringic acid and the SYA@MIL-100(Fe)-10% formulation following oral and intraperitoneal administration.

Test Compound	Biological Sample	Route	AUC_0–72_ *	AUC_0–∞_ *	C_max_ **	T_max_ ***	T_1/2_ ***
Syringic Acid	Blood	Oral	1419.00 ± 142.15	1460.37 ± 143.84	2.34 ± 0.19	66.78 ± 7.56	118.77 ± 999.64
		Intraperitoneal	4368.33 ± 489.25	5460.84 ± 964.81	2.55 ± 0.04	55.97 ± 2.11 ^‡^	999.64 ± 410
	Liver	Oral	32,000.33 ± 3544.16 ^‡^	47,401.91 ± 8515.77 ^‡^	16.28 ± 3.54	515.98 ± 4.44 ^‡^	2288.81 ± 812.66
		Intraperitoneal	102,784.67 ± 1510.75	166,522.15 ± 34,487.20	40.13 ± 0.56	24.70 ± 0.21	1852.40 ± 1105.09
	Kidney	Oral	77,153.33 ± 2531.03	78,035.27 ± 2458.81 ^‡^	105.36 ± 9.79	28.21 ± 0.33	37.56 ± 4.69
		Intraperitoneal	321,104.67 ± 11,949.32	808,296.73 ± 429,472.17	218.21 ± 8.73	24.15 ± 0.96	792.21 ± 153.25
SYA@MIL-100(Fe)	Blood	Oral	7221.33 ± 97.63 ^‡^	7634.00 ± 97.60 ^‡^	2.60 ± 0.01	52.02 ± 1.34	186.15 ± 2.62
		Intraperitoneal	31,297.33 ± 661.33 ^‡^	42,846.93 ± 3703.76 ^‡^	16.62 ± 0.44 ^‡^	26.72 ± 1.03	997.69 ± 377.83
	Liver	Oral	16,754.33 ± 2268.79	17,264.74 ± 2353.25	16.89 ± 1.84	35.46 ± 4.39	110.08 ± 11.75
		Intraperitoneal	719,560.00 ± 14,449.94 ^‡^	2,893,510.76 ± 1,716,470.52	245.65 ± 10.86 ^‡^	110.54 ± 4.25 ^‡^	8312.26 ± 6290.46
	Kidney	Oral	88,202.33 ± 4855.64	93,818.14 ± 4364.84 ^‡^	127.28 ± 5.96	94.54 ± 5.27 ^‡^	236.36 ± 71.08 ^‡^
		Intraperitoneal	1,911,750.00 ± 76,232.91 ^‡^	21,513,220.82 ± 16,644,903.64	669.96 ± 26.14 ^‡^	94.96 ± 1.36 ^‡^	28,587.55 ± 24,474.46

*—unit of area under the curve is mg min mL^−1^ (for blood sample) and ug min g^−1^ (for kidney and liver samples); **—unit of C_max_ is mg mL^−1^ (for blood sample) and ug g^−1^ (for kidney and liver samples); ***—unit of T_max_ and T_1/2_ is min; ^‡^—significant differences at *p*-value < 0.05 (syringic acid vs. SYA@MIL-100(Fe)-10%). Data presented is mean ± S.E.M (n = 3 per data point).

## Data Availability

The raw data supporting the conclusions of this article will be made available by the authors on request.

## Supplementary Materials

The following supporting information can be downloaded at: https://www.mdpi.com/article/10.3390/pharmaceutics18030309/s1, Figure S1: Synthesized MIL-100(Fe)-XX% (left to right): 10%, 20%, 30%, 40%, 50% and 100% of syringic acid as organic linker; Figure S2: Image comparing the (A) MIL-100(Fe) and (B) MIL-100(Fe)-10%.

## Abbreviations

The following abbreviations are used in this manuscript:

MIL	Materials of Institut Lavoisier
MOF	Metal organic framework
AUC	Area under the curve
C_max_	Maximum concentration
T_max_	Time to reach maximum concentration
T_1/2_	Half-life
SD	Sprague Dawley
SYA@MIL-100(Fe)	Syringic acid-loaded MIL-100(Fe)
