# Supplementary material for: Tailoring Syringic Acid–Trimesic Acid Mixed-Linker MIL-100(Fe): Evaluation of Drug-Loading Capacity, Bioavailability, and Toxicity
Source: Pharmaceutics. 2026 Feb 28;18(3):309. doi: 10.3390/pharmaceutics18030309 (PMC13028778; doi:10.3390/pharmaceutics18030309)
Supplement: Supplementary file 1 [file pharmaceutics-18-00309-s001.zip › pharmaceutics-4077508-supplementary.pdf]

Article

Tailoring Syringic Acid–Trimesic Acid Mixed-Linker MIL-100(Fe): Evaluation of Drug-Loading Capacity, Bioavailability, and Toxicity

Joshua H. Santos [1,2,*], Hannah Jean Victoriano [2], Mary Sepulveda [2], Hung-En Liu [3], Shierrie Mae N. Valencia [2], Rikkamae Zinca Marie L. Walde [2], Emelda A. Ongo [1,2] and Chia-Her Lin [4,*]

1 Department of Science and Technology—Central Office, General Santos Avenue, Upper Bicutan, Taguig City 1631, Philippines
2 Department of Science and Technology, Industrial Technology Development Institute, General Santos Avenue, Upper Bicutan, Taguig City 1631, Philippines
3 Department of Chemistry, National Taiwan Normal University, No. 162, Section 1, Heping E Rd, Da'an District, Taipei City 106, Taiwan
4 Department of Chemistry, National Tsing Hua University, 101, Section 2, Kuang-Fu Road, East District, Hsinchu 300044, Taiwan
* Correspondence: jhsantos120@gmail.com (J.H.S.); chiaher@mx.nthu.edu.tw (C.-H.L.); Tel.: +632-88-37-2071 to 82 local 2276 (C.-H.L.)

Supplementary Data for Synthesis of MIL-100(Fe)

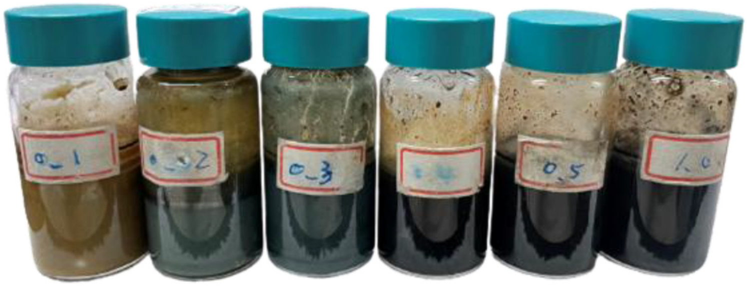

Figure S1. Synthesized MIL-100(Fe)-XX% (left to right): 10%, 20%, 30%, 40%, 50% and 100% of syringic acid as organic linker.

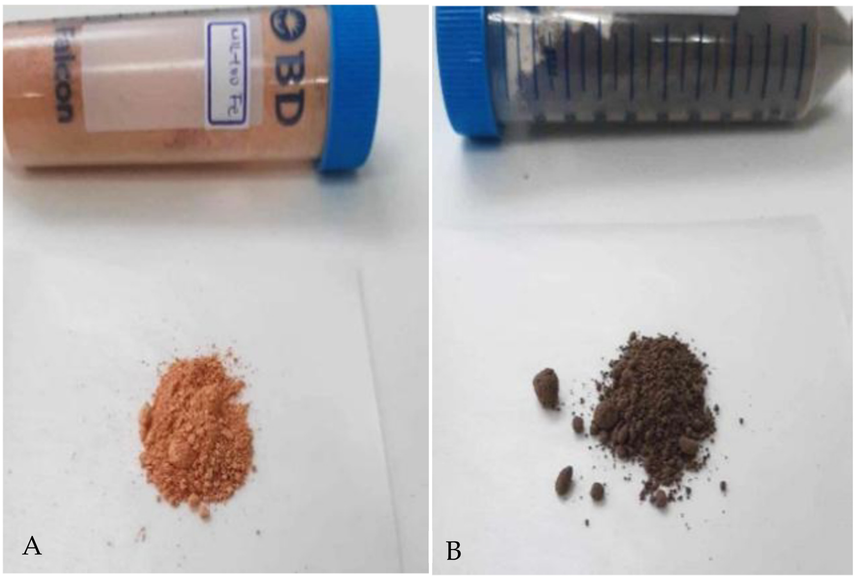

Figure S2. Image comparing the (A) MIL-100(Fe) and (B) MIL-100(Fe)-10%
